# Laser solid-phase synthesis of graphene shell-encapsulated high-entropy alloy nanoparticles

**DOI:** 10.1038/s41377-024-01614-y

**Published:** 2024-09-26

**Authors:** Yuxiang Liu, Jianghuai Yuan, Jiantao Zhou, Kewen Pan, Ran Zhang, Rongxia Zhao, Lin Li, Yihe Huang, Zhu Liu

**Affiliations:** Research Centre for Laser Extreme Manufacturing, Ningbo Institute of Materials Technology and Engineering, Chinese Academy of Sciences, Ningbo, 315201 China

**Keywords:** Physics, Lasers, LEDs and light sources

## Abstract

Rapid synthesis of high-entropy alloy nanoparticles (HEA NPs) offers new opportunities to develop functional materials in widespread applications. Although some methods have successfully produced HEA NPs, these methods generally require rigorous conditions such as high pressure, high temperature, restricted atmosphere, and limited substrates, which impede practical viability. In this work, we report laser solid-phase synthesis of CrMnFeCoNi nanoparticles by laser irradiation of mixed metal precursors on a laser-induced graphene (LIG) support with a 3D porous structure. The CrMnFeCoNi nanoparticles are embraced by several graphene layers, forming graphene shell-encapsulated HEA nanoparticles. The mechanisms of the laser solid-phase synthesis of HEA NPs on LIG supports are investigated through theoretical simulation and experimental observations, in consideration of mixed metal precursor adsorption, thermal decomposition, reduction through electrons from laser-induced thermionic emission, and liquid beads splitting. The production rate reaches up to 30 g/h under the current laser setup. The laser-synthesized graphene shell-encapsulated CrMnFeCoNi NPs loaded on LIG-coated carbon paper are used directly as 3D binder-free integrated electrodes and exhibited excellent electrocatalytic activity towards oxygen evolution reaction with an overpotential of 293 mV at the current density of 10 mA/cm^2^ and exceptional stability over 428 h in alkaline media, outperforming the commercial RuO_2_ catalyst and the relevant catalysts reported by other methods. This work also demonstrates the versatility of this technique through the successful synthesis of CrMnFeCoNi oxide, sulfide, and phosphide nanoparticles.

## Abstract (web summary)

We demonstrate a simple and versatile method to synthesize graphene shell-encapsulated high-entropy alloy nanoparticles through laser irradiation of mixed metal precursors in solid-phase, offering excellent electrocatalytic activity and stability.

## Graphical abstract

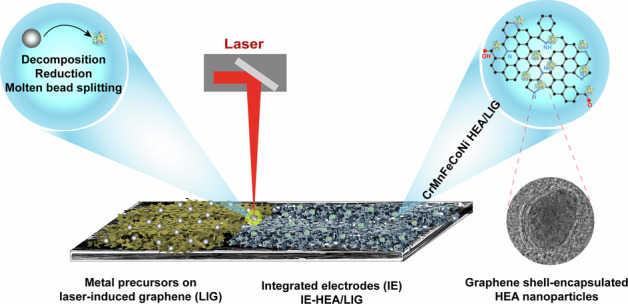

## Introduction

High-entropy alloys (HEAs) contain five or more principal elements in equimolar or near-equimolar ratios. When the size of HEAs decreases to nanoscale, such as nanoparticles, the high specific surface area, quantum size effect, strong synergistic interaction, and lattice distortion make them an ideal platform for a variety of surface reactions, showing great promise for a wide range of emerging energy-related applications, particularly in the field of catalysis^1–4^.

There are two categories of methods for the preparation of high-entropy alloy nanoparticles (HEA NPs), generally classified as the “top-down” physical methods by crushing bulk materials and the “bottom-up” chemical methods by reducing metal salt precursors. The HEA NPs prepared by ball milling usually display a large distribution in the composition^5^. Wet chemical synthesis can effectively control the composition, phase structure, and particle size of the target elements^6–8^. However, due to the huge differences in the chemical and physical properties of metal salt precursors (e.g., redox potentials of individual components, thermal decomposition temperature, etc.), it is difficult to achieve simultaneous decomposition or reduction^8^. Therefore, it is prone to producing alloy nanoparticles with severe phase separation^8^, which impedes the HEA material design, mechanism studies and performance optimization.

In recent years, advances in the exploration of rapid and controllable methods for HEA NPs without phase separation have been made^9–13^. These methods require high reaction temperatures, and rapid cooling to maintain a high-entropy state at room temperature. As a result, the preparation of high-entropy nanoparticles with a single solid solution can be achieved in a non-equilibrium state by suppressing the formation of secondary phases. In 2018, a carbothermal shock method was reported by Yao et al. to alloy up to eight elements into HEA NPs with the designated composition and size^9^. This method involves a 55-ms heating of mixed metal precursors on carbon supports at a peak temperature of 2000 K in argon and cooling at a cooling rate of ∼10^5^ K/s. The high temperature ensures uniform mixtures of multiple elements by fission/fusion and catalytically driven particle dispersion mechanisms. This work has made an important step in the rapid synthesis of high-entropy alloy nanoparticles. However, this method can only be applied to conductive, surface-oxidized carbon support materials. Since then, more work has been reported on the rapid synthesis of HEA NPs within milliseconds to seconds, including fast-moving bed heating^10^, electrical sparkling^11^, Joule heating^12^ and microwave heating^13^. For example, the microwave heating method with a heating temperature of ∼2000 K within 5 s, and a cooling rate of ∼10^4 ^K/s, resulted in the decomposition of the precursors into liquid metal, to form PtPdFeCoNi HEA-NPs, with an average particle size of ∼12 nm and uniform elemental mixing^13^. These methods require fast heating and cooling rates, complex heating equipment, and good matrix conductivity or microwave absorption capacity. Therefore, a more cost-effective, versatile, and adaptable technology for large-scale manufacturing of high-entropy alloy nanomaterials is needed to be developed for practical applications.

Laser heating is mainly based on the absorption of laser beams by materials, which can produce a very high temporal temperature and rapid cooling on the surface of the materials in a controllable manner. This offers an alternative way for the rapid synthesis of HEA nanomaterials. Pulsed laser ablation from a solid target immersed in liquid has been considered as a green physical route for scalable nanoparticle fabrication^14–16^. In 2019, Waag et al. reported the fabrication of isolated, colloidal CoCrFeNiMn NPs by picosecond-pulsed laser ablation of a solid CoCrFeNiMn HEA target immersed in a flow cell^16^. This is a typical “top-down” method, which requires the target with the same composition as the HEA NPs to be produced. In 2022, Yao and Zou et al. extended the laser ablation in liquid method, by using a 5 ns pulsed laser to ablate the mixed metal precursor immersed in liquid, leading to synthesizing a series of high-entropy alloy and ceramic nanoparticles loading on various substrates^17^. In addition, Jiang et al. reported the direct fabrication of HEA NPs on carbonaceous support under atmospheric conditions via nanosecond pulsed laser reduction of powdery metal salt precursors in a container, based on laser-induced thermionic emission mechanism^18^, further demonstrating the potential of laser technology in the field of HEA NPs fabrication. In 2023, Li et al. reported another method using a continuous wave 532 nm wavelength laser, under a nitrogen/ambient atmosphere, to synthesize high-entropy alloy, oxide, and nitride nanoparticles on porous carbon nanofiber and graphene oxide-coated glass substrates, and also investigated the laser annealing induced phase transformation behaviors^19^. To date, the laser preparation of HEA NPs is in an early stage. When a laser beam irradiates on a material surface, the temperature distribution along the depth decreases. In addition, due to the large differences in the physiochemical properties of metal salt precursors in terms of decomposition temperature, melting temperature of corresponding metals, etc., the underlying mechanisms involved in laser synthesis are rather complicated. On the other hand, we still need to further explore laser technology to achieve a highly productive and cost-effective laser synthesis method that can be applied for scalable manufacturing of HEA NPs loaded on various material supports.

In this work, we report a laser solid-phase synthesis of HEA NPs on 3D porous laser-induced graphene (LIG), denoted by HEA/LIG. The focus of the work was placed on the mechanism of the laser synthesis in the consideration of laser beam interaction with metal salt precursors and carbon-based materials. We intended to gain an understanding of the HEA NP formation through thermodynamic modeling and experimental measurements in terms of temperature variation and electron emission under laser irradiation, to provide a theoretical basis and a reliable technical route for the rapid laser synthesis of HEA NPs. We chose CrMnFeCoNi, as CrMnFeCoNi is relatively a well-researched alloy among the HEAs, in which both miscible (Cr/Fe) and immiscible metal pairs (Mn/Co) are present. We used the laser-induced 3D porous graphene from polybenzimidazole (PBI) to serve as the host to adsorb mixed metal salt precursors and then subjected it to laser irradiation using a Nd:YAG laser with a wavelength of 1064 nm and a pulse width of 30 ns. The effects of laser fluences and concentrations of the mixed metal precursors on the size of HEA NPs were investigated. We studied the synthesis mechanism through laser-induced photothermal decomposition of the metal precursors in combination with laser-induced thermionic emission reduction. Compared with the laser ablation in liquid, laser solid-phase synthesis offers a new route of HEA NP synthesis which can be characterized by fine controllability, no post-treatment, simple and easy operation, scalability and low cost, and high production rate of 29.6 g/h with the current laser setup. The laser-synthesized CrMnFeCoNi HEA NPs/LIG can be assembled directly to integrated electrodes (IEs), which is denoted by IE-HEA/LIG. The IE-HEA/LIG exhibited higher catalytic activity and exceptional stability toward oxygen evolution reaction (OER) over the commercial RuO_2_/C catalyst, manifesting the potential of the method to produce HEA NPs as a heterogeneous catalyst. This technique has been further extended to the synthesis of CrMnFeCoNi oxide, sulfide, and phosphide, demonstrating its versatility and an industrially viable solution to the challenge of rapid synthesis of HEA and other nanoparticles.

## Results

**Fig. 1:**
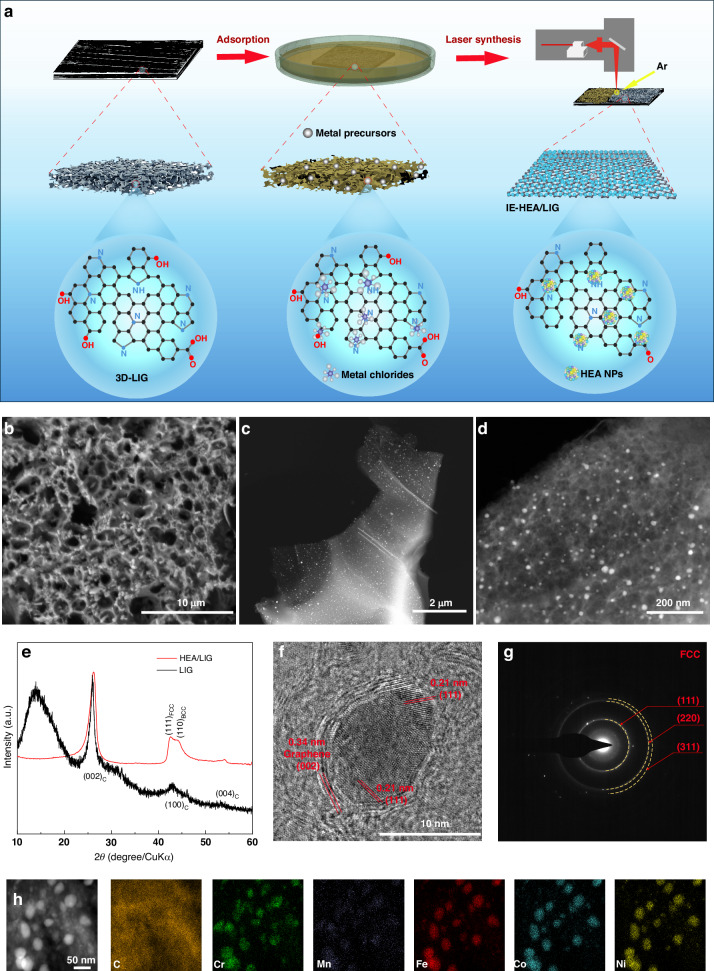
Laser solid-phase synthesis procedure and characterization of HEA NPs. **a** Schematic diagram of the laser synthesis procedure for the CrMnFeCoNi HEA nanoparticles and IE-HEA/LIG. **b** FE-SEM images of the metal precursors on LIG. **c**, **d** TEM images of the HEA NPs. **e** XRD spectra of the LIG and HEA/LIG. **f**, **g** HR-TEM image of HEA nanoparticles on LIG and the SAED pattern (scale bar = 10 nm in (**f**)). **h** STEM elemental mappings for the HEA/LIG (scale bar = 50 nm)

The laser synthesis procedure is illustrated in Fig. 1a. Firstly, metal chlorides (CrCl_3_·6H_2_O, MnCl_2_, FeCl_3_, CoCl_2_·6H_2_O and NiCl_2_·6H_2_O) with equal-molar ratios were dissolved in deionized water. Then, the LIG-coated carbon paper samples, as evidenced by the Raman spectrum (Fig. S1), were immersed in the mixed solutions with variable concentrations (1, 5, 10, 15, or 20 mM). The preparation of the LIG was described in our previous work^20^ and the Experimental Section. After drying in a vacuum oven at 80 °C for 30 min, the samples were subjected to laser irradiation, using an Nd: YAG laser with a wavelength of 1064 nm and a pulse width of 30 ns, under an Ar atmosphere via rapid scanning at the scanning rate of 2000 mm/s. After the laser treatment, the final samples (HEA/LIG and IE-HEA/LIG) were obtained for further investigations (as detailed in the Experimental Section).

### Characterization of laser-synthesized CrMnFeCoNi NPs

The mixed metal precursor before laser irradiation shows a crystalline structure that does not belong to any of the five metal chlorides (Fig. S2a). The metal precursors are distributed on the surface and inside the pores of the 3D-LIG (Fig. 1b). Transmission electron microscope (TEM) investigations of the metal precursors loaded on LIG reveal that the metal precursors are present in the form of irregular particles, around 50 nm and densely loaded on the LIG (Fig. S2b and S2c). The selected-area electron diffraction (SAED) pattern (Fig. S2d) shows that the metal precursors are characterized by several diffraction rings of (001), (201), (140), and (402). Energy-dispersive X-ray spectroscopy (EDS) analysis shows all five elements with relatively homogeneous distribution in the particle in Fig. S2e. After the laser treatment, the metal precursors are converted to the HEA nanoparticles, as seen in Fig. 1c, d. The nanoparticles are highly dispersed on the LIG and the particles are uniform in size. The phase composition of the synthesized HEA nanoparticles was examined by X-ray diffraction (XRD) (Fig. 1e). Only the characteristic peak of C was detected for the LIG samples, while the diffraction peak at 43° appears on the HEA/LIG samples, corresponding to the (111) orientation of the FCC structure. In addition, the high-resolution TEM (HR-TEM) image (Fig. 1f) and the SAED pattern (Fig. 1g) show that the nanoparticles are polycrystalline, indexed to (111), (220), and (311), corresponding to the d-spacings of 0.21, 1.94 and 1.75 nm, respectively. The SAED patterns are in agreement with the XRD results, revealing that the crystal structure of CrMnFeCoNi HEA nanoparticles is FCC solid solution. By analyzing the TEM images and EDS elemental mappings (Fig. 1h), all five elements relatively homogeneously are distributed within the CrMnFeCoNi HEA nanoparticles, in which the contents of the Fe, Co and Ni elements are higher than those of the Cr and Mn (Fig. S3), benefiting from their closer reduction potential and fewer valence electrons that form stable compounds while Mn is expected to be partially vaporized during the laser synthesis due to its higher vapor pressure. Moreover, the boiling temperature of 2334 K (Table S3) of Mn is the lowest and close to the highest temperature induced by laser, thus the Mn element has a relatively high possibility to evaporate.

The HRTEM images in Fig. 1f and Fig. S4b–d, also show that the HEA nanoparticles are partially embraced by several graphene layers. This is similar to carbon-shell-encapsulated nanoparticles that were reported and achieved by Sharma et al. for improved catalytic durability on electrochemical reactions^21,22^.

**Fig. 2:**
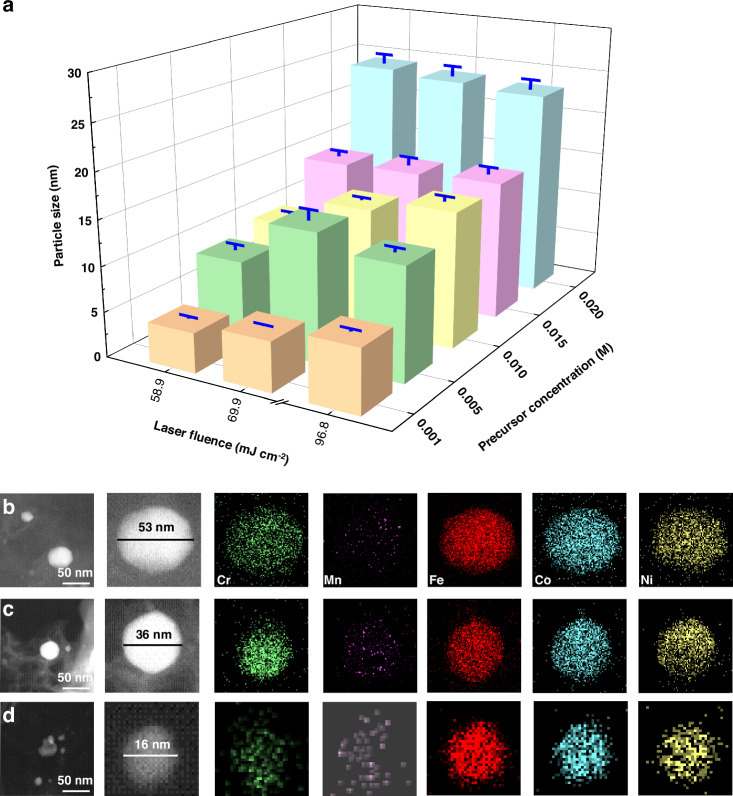
Particle size and elemental distribution of HEA NPs. **a** Particle size varying with laser fluences and precursor concentrations. **b**–**d** EDS elemental mappings of the HEA/LIG for different particle sizes

Figure 2a and Fig. S5 show the variation of the size of CrMnFeCoNi HEA nanoparticles with the concentrations of the metal precursors and laser fluences, with statistical particle size distributions. The particle size becomes bigger with increasing the precursor concentrations, which is more pronounced at lower laser fluences. When the precursor concentration is lower than 0.01 M, the particle size increases with laser fluences. Further increasing the concentrations, the particle size shows insignificant changes with increasing the laser fluences. Overall, the nanoparticles have a uniform size (about 15 nm on average) when the precursor concentration is between 0.005 M and 0.015 M while the average size is significantly increased, reaching up to 23 nm at 0.02 M precursor concentration. Figure 2b–d shows the EDS elemental mappings of the three typical CrMnFeCoNi HEA nanoparticles in different sizes, giving evidence of no apparent phase separation.

### Laser synthesis mechanisms

In order to investigate the laser synthesis mechanism, it is necessary to consider the adsorption behavior of the metal precursors on LIG, in terms of carbon moieties and precursor concentrations. As has been reported before^20^, the LIG has special chemical (surface charge and functional groups, et, al) and physical (surface area, pores, defects, et, al) properties, in addition to the 3D porous structure. In this work, the surface elemental composition and chemical states were further characterized by XPS in Fig. S6. The results show the N, O, and C elements and carbon moieties including C = C, C-C, C − N, -C = O, O = C-OH, and O-C = O existing on the LIG. Through comparison of the integrated intensity of C, N, and O, the atomic percentages of the three elements are 92.8, 4.97, and 2.23%, respectively. Therefore, the LIG as a host offers abundant adsorption sites to anchor the metal precursors. The adsorption occurs via the mechanisms of complexation^23^, electrostatic interactions^24^, π-cations, and ion exchange^25^. Based on the SEM observation, the adsorption of the metal chlorides on the LIG is in the form of nanoparticles which distribute sparsely and evenly on the LIG, and the particle size is dependent on the concentrations of the metal precursors. At a low concentration such as 0.002 M, the metal chloride monomers (several metal cations and Cl^−^ anions) are attracted or dragged by the active adsorption sites, and the adsorbed metal chlorides are in the sub-nanometer scale due to the low chemical potential and the fast equilibrium between adsorption and desorption of the metal ions. With increasing concentrations, the metal precursors tend to accumulate into larger particles on the LIG (Fig. S7).

To understand the interaction between the laser beam and metal precursors as well as the LIG, light transmission spectra of the metal precursors and the metal precursor-adsorbed LIG on carbon paper were measured using UV-Vis Spectroscopy (Fig. S8). It reveals that the light transmittance at the wavelength of 1064 nm for the mixed metal salts is 96.77% while it is 0.005% for the metal salt-adsorbed LIG on the carbon paper, suggesting that the metal salts could be considered to be almost transparent to the laser beam, but well absorbed by the LIG on the carbon paper. Therefore, it is likely that upon the laser irradiation, the laser beam goes through the metal precursors to interact with the LIG and generates heat via a photothermal effect, and then the heat conducts to the metal precursors to cause its temperature rise.

**Fig. 3:**
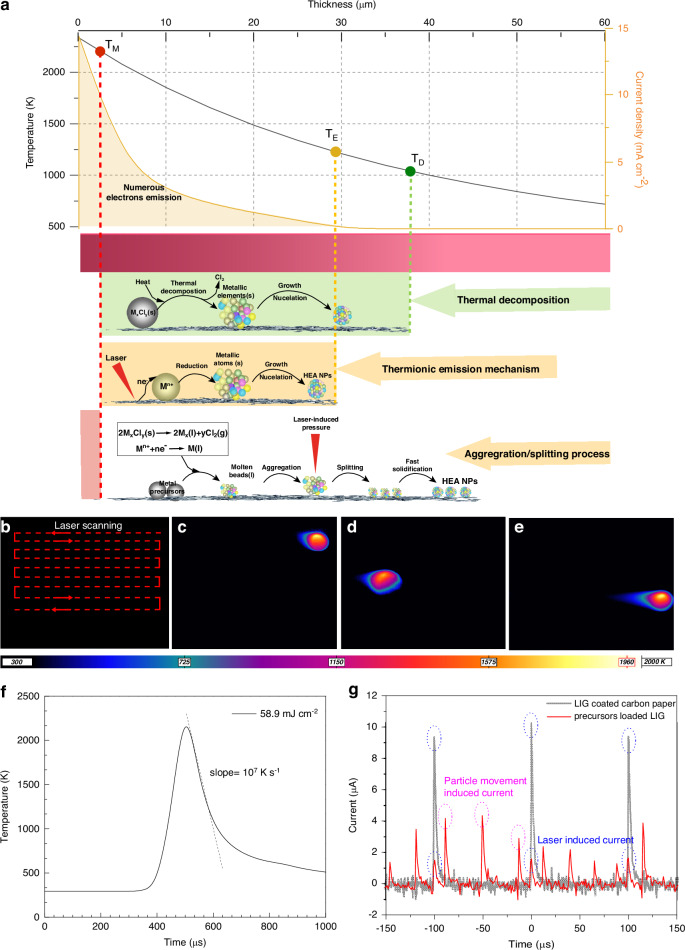
Laser solid-phase synthesis mechanisms. **a** Schematic diagram of the laser synthesis mechanisms. **b** Laser scanning method. **c–e** Temperature recorded (K) in three representative locations during laser irradiation. **f** Simulated temperature evolution of one point under laser irradiation. **g** Measured emission current of the LIG and precursors loaded LIG under laser irradiation. Noting: the laser synthesis parameters are of 2000 mm/s scanning rate, 10 kHz pulse repetition rate, 430 μm spot diameter, and 58.9 mJ/cm^2^ laser fluences for experiments and simulation in (**b**–**g**)

The temperature was experimentally recorded and simulated (Fig. 3b–e and Figs. S9, S10). Based on the experimental measurements, at a laser fluence of 58.9 mJ/cm^2^, a scanning rate of 2000 mm/s and a repetition rate of 10 kHz, the localized surface temperature reaches 1960 K. To reveal the thermal evolution, ABAQUS software was used to simulate the temperature dynamics at the same laser operating condition. The simulated temporal thermal profile on the surface in Fig. 3f shows a heating time of 100 μs to reach up to 2381 K, and a cooling rate of 10^7 ^K/s. In addition, it is known that when a laser beam irradiates on a solid substrate, the laser energy can be converted into heat and the heat will conduct to raise the substrate temperature locally and along the depth of the substrate, the temperature decreases gradually. From the simulation result (Fig. S10b), it can be seen that the temperature decreases sharply along the depth of the carbon support. Therefore the surface and the interior of the LIG with adsorbed metal precursors experience different thermal evolution.

As shown in Table S2, the thermal decomposition of the five metal salts occurs at different temperatures. Figure 3a shows the temperature profile along the depth. Once the thermal decomposition temperature (T_D_) of the metal precursor is reached but below the melting temperature (T_M_) of the corresponding metals, the thermal decomposition of the mixed metal salt particles adsorbed on the LIG occurs accompanied by gas release, and then each CrMnFeCoNi nanoparticle with homogeneous composition is generated by nucleation and growth of the species from the decomposition of each mixed metal salt particle. This particle is likely to be at the site of the original metal salt particle (the light green zone in Fig. 3a). When the temperature is above T_M_ while below the boiling temperature (T_B_) of metals, the metallic elements are in the liquid phase to form molten beads. Since metals are nonwetting with carbon, these liquid metal beads tend to aggregate and grow bigger to minimize their surface energy at high temperatures^26^. However, our observation differs from this behavior, i.e., the size of the CrMnFeCoNi HEA NPs on the surface is on a similar length scale as the nanoparticles found elsewhere. This might be attributed to the laser-induced high pressure^27^, which impacts to the molten beads to cause splitting up the molten beads into small-sized droplets, followed by rapid solidification to form smaller particles (the light red zone in Fig. 3a).

In addition to the thermal decomposition process described above, there is another process contributing to the formation of CrMnFeCoNi HEA NPs. It is known that graphene is prone to be excited by laser to cause thermionic emission^28^. When a laser beam is absorbed by graphene, electrons from the valence band are excited to the conduction band, and population inversion can be achieved and maintained. Then, hot electrons obtained with sufficient energy can be ejected from the graphene and become free electrons through Auger-like pathways^28^. The emission current can be estimated by Richardson Law^29^, expressed by

$${\boldsymbol{i}}={\boldsymbol{S}}{\times {\boldsymbol{A}}}_{{\bf{LIG}}}{\times {\boldsymbol{T}}}^{{\bf{2}}}{\times {\bf{e}}}^{(-{\rm{\varnothing }}/{{\bf{k}}}_{{\boldsymbol{0}}}{\boldsymbol{T}})}$$

Where *i* is the emission current, *S* is the emission area, *A*_LIG_ is Richardson’s constant of the LIG (120 A · cm^−2 ^· K^−2^), ɸ is the work function of the LIG (4.39 eV), k_0_ is Boltzmann’s constant (8.617 × 10^−5 ^eV/K), and *T* is the local temperature.

To verify the emission of electrons upon the laser irradiation on the LIG, a house-made device was used to detect the ejected electrons from the LIG under the laser irradiation by monitoring the current flow in a circuit (Fig. S11a, b). The results show that at a bias voltage of 3 kV, the emission current reaches 0.15 μA under ambient conditions (Fig. 3g). The free electrons play an important role in the formation of CrMnFeCoNi HEA NPs by reducing the metal ions into metal atoms. The calculated current density at different temperatures is plotted in Fig. 3a (the yellow curve). When the temperature is below T_E_, the current density is considered to be negligible. Towards the surface, the current density increases. At the temperature between T_E_ and T_M_, the electrons emitted from the LIG support can be captured by the metal ions adsorbed on LIG, leading to the reduction of the metal ions, followed by nucleation and growth to homogeneous nanoparticles, which are also likely to sit on the same site of the original metal salt particles (the light yellow zone in Fig. 3a). When the temperature is above the melting points of the metallic elements, but lower than the boiling points, T_B_, the metallic elements are in a liquid phase, forming molten beads which experience the same aggregation and splitting process as discussed above.

As described earlier, the size of the CrMnFeCoNi HEA nanoparticles is strongly dependent on the metal precursor concentrations. This is the result of the increased size of the mixed metal precursor particles with increased concentrations of the metal precursor. On the other hand, the size of the CrMnFeCoNi HEA nanoparticles increases with the laser fluence, which is more pronounced at lower laser fluences. As shown in Fig. 2a, when the concentration of the metal precursors reaches 0.015 mM, the size of the nanoparticles is only slightly increased. This could be explained based on the mechanism described earlier, as follows. With the increasing laser fluence, the temperature above T_M_ has a bigger spatial and temporal region (the light red zone becomes bigger in Fig. 3), thus the aggregation of molten beads should become more easily to form even bigger beads. However, the experimental observation confirmed the uniformity of the nanoparticle size, attributing to the more effective splitting driven by the higher laser-induced pressure with laser fluences.

### Application in electrocatalysis

Over the last few years, there has been a significant increase in the applications of HEAs in electrocatalysis^30–32^. The emergence of HEAs has shown their unique capability of overcoming the limitations of transition metal alloys in acidic or alkaline environments due to their remarkable resistance to corrosion and toxicity during the electrochemical reaction process^32^. Currently, most electrocatalysts are powder-based. Electrode preparation follows a coating route involving the use of low-conductive polymeric binders, which may elevate resistance levels, block active sites, impede mass transport, and consequently lead to a degradation in catalytic performance^33,34^. Additionally, continuous gas evolution during electrochemical reactions may also result in detachment of the electrocatalysts. Therefore, it is highly desirable to develop a method to fabricate binder-free integrated electrocatalytic electrodes that is time-efficient, versatile, green, low-cost, and suitable for large-scale production.

**Fig. 4:**
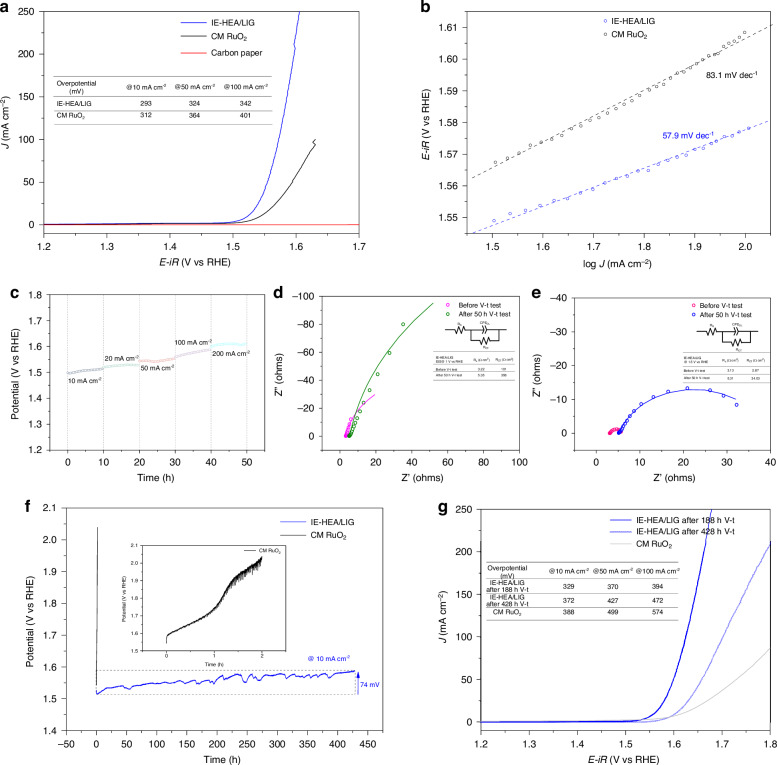
OER performance of IE-HEA/LIGs in 1 M KOH electrolyte. **a** LSV plots, **b** Tafel slopes, **c** V-t curves at 10, 20, 50, 100, 200 mA/cm^−2^, **d**, **e** Nyquist plots at 1 V and 1.5 V vs. RHE, **f** Long-term stability at 10 mA/cm^−2^, **g** LSV plots after long-term V-t tests

In this work, the laser-synthesized CrMnFeCoNi HEA nanoparticles on LIG-coated carbon paper were used directly as electrocatalytic electrodes for OER to evaluate its electrocatalytic activity and stability in 1 M KOH alkaline media. The linear sweep voltammogram (LSV) curves of the purchased carbon paper, commercial RuO_2_, and IE-HEA/LIG sample after ohmic-drop correction are shown in Fig. 4a. The carbon paper shows negligible current change under the applied voltage from 1.2 to 1.7 V vs RHE. The IE-HEA/LIG exhibits enhanced OER activity compared to the commercial RuO_2_. The overpotentials of IE-HEA/LIG to drive anodic current densities of 10, 50, and 100 mA/cm^2^ are 293, 324, and 342 mV, respectively, which are 312, 364, and 401 mV for the commercial RuO_2_. Figure 4b shows the OER kinetics derived from LSV of commercial RuO_2_ and the IE-HEA/LIGs. The Tafel slopes are 83.1 and 57.9 mV/dec for commercial RuO_2_ and IE-HEA/LIG, respectively, signifying the superior OER rate of the IE-HEA/LIG. The enhanced activity and kinetic of IE-HEA/LIG derive from the solid-solution structure and multiple active sites of the HEA nanoparticles, bringing about more OER paths and lower energy barriers for such reactions.

Figure 4c shows the chronopotentiometric (V-t test) results. The test was conducted on the IE-HEA/LIG samples in a continuous mode for 10 h at 10, 20, 50, 100, and 200 mA/cm^2^, respectively. The potentials remain stable at each current density stage and the IE-HEA/LIG shows excellent stability at high current densities. Electrochemical Impedance Spectroscopy (EIS) measurements were performed on the IE-HEA/LIGs before and after the 50 h stability test at an applied potential of 1 and 1.5 V vs. RHE, respectively, to measure the charge transfer resistance (*R*_CT_) and evaluate the catalyst/electrolyte interface characteristics. The Nyquist plots and fitting results of the samples in Fig. 4d, e indicate the *R*_CT_ values of the samples vary with the applied potential and the experimental conditions. Applied with 1 V vs. RHE, the samples have no external force to drive the OER and the electrochemical reaction rate is rather slow, resulting in big *R*_CT_ (Fig. 4d) for the sample before and after the V-t test. By comparison, OER is triggered and oxygen bubbles are continuously released on the sample when applied with the potential of 1.5 V vs. RHE, thus the *R*_CT_ values are small and the electrochemical reaction is running fast. Accompanied by oxygen bubbles, the HEA is oxidized (Fig. S12, Supporting Information). The oxides change the dielectric and electric double-layer properties of the solid/liquid interface, resulting in a small RC constant and a curved Nyquist plot in Fig. 4e. For the two different applied potentials, the *R*_CT_ after V-t test is consistently bigger than the values before V-t test. This result can probably be attributed to the oxidation of the HEA catalyst during V-t test. The oxides and hydroxides play a role in increasing the impedance of the OER process. However, they do not suffocate the HEA catalyst for producing oxygen.

To investigate the stability of the IE-HEA/LIGs, long-life V-t tests were conducted and the results are shown in Fig. 4f. The V-t result of commercial RuO_2_ was also recorded as a comparison. In Fig. 4f, at 10 mA/cm^2^, the potential of commercial RuO_2_ increases sharply within 2 h and reaches over 2 V. Whereas, for IE-HEA/LIGs, the potential has a small rise of about 74 mV for 428 h, showing its excellent stability. The increase of the potential likely originates from the developed oxides/hydroxides and the corresponding electrochemical impedance. Also, the LSV curves of IE-HEA/LIGs after 188 and 428 h V-t tests (Fig. 4g) show that the overpotentials decay reasonably. However, the overpotential at 10 mA/cm^2^ of IE-HEA/LIGs after 428 h V-t test is 372 mV which is still smaller than that (388 mV) of commercial RuO_2_ after only 2 h test. Consistently, the overpotential of 372 mV is approximately the sum of the initial overpotential of 293 mV before V-t test and the elevated 74 mV during V-t test. Experimentally, we observed that accompanied by rapid oxygen release, the commercial RuO_2_ sample disintegrated within 2 h and black pieces of carbon paper floated on the top of the electrolyte. This phenomenon can also be observed on the IE-HEA/LIGs after 200 h, just with less debris. The disintegration of carbon paper support can be attributed to the rapid oxygen gas evolution rate during electrolysis.

Overall, the OER performance of the IE-HEA/LIG in this work is excellent, outweighing most of the non-precious HEA catalysts reported up to date (Table S4). It is worth noting that the stability of the laser-synthesized IE-HEA/LIG outperforms most of the precious and non-precious HEA catalysts.

## Discussion

As described above, during laser irradiation, the conversion of the HEA NPs from the mixed metal precursors adsorbed on LIG involves several processes, including thermal decomposition, metal ion reduction, metallic element melting, molten bead fusion, and fission, depending on the temperature experience. The laser synthesis in this work is based on solid-phase, which is different from liquid-phase synthesis. As the mixed metal precursor is adsorbed on the 3D porous LIG structure and dried before laser irradiation, the size of the nanoparticles can be well controlled by the laser fluence, and the concentration of the precursor due to the limited mobility of the reduced metal species and subsequently the nanoparticles.

The laser-synthesized CrMnFeCoNi nanoparticles are embraced by several graphene layers, forming graphene shell-encapsulated nanoparticles. This might be caused by the bending of graphene layers subjected to laser heating, which was also reported in the work on graphene nanosphere formation by microwave heating^35^. Embracing the metal nanoparticles by graphene layers is beneficial to the dispersion of the nanoparticles by preventing nanoparticles from aggregation. On the other hand, the analysis of the LIG indicates that the LIG is characterized by a high density of edge planes (Fig. S4c, Supporting Information), which are highly defective, offering preferred sites to immobilize the HEA nanoparticles (Fig. S4d, Supporting Information). This phenomenon was also reported in the synthesis of metallic nanoparticles on carbon supports through the Joule heating method^36^. Both characteristics of the laser-synthesized CrMnFeCoNi HEA nanoparticles on LIG support described above offer a reinforced effect on the nanoparticles with strong adhesion to the LIG, which might contribute to the high stability of the CrMnFeCoNi HEA nanoparticles catalysts in catalytic applications.

The electrocatalytic performance described in this work is superior to most of the non-precious HEA catalysts reported up to date. Particularly, the stability of the laser-synthesized IE-HEA/LIG outperforms most of the precious and non-precious HEA catalysts. This might be explained in consideration of the following aspects. Firstly, the laser-synthesized CrMnFeCoNi HEA nanoparticles are the form of the graphene shell-encapsulated. This hybrid structure offers benefits for enhancing the electronic transport from the LIG to the active sites, and the incomplete wrap (Fig. 1f and Fig. S4a, b) benefits the exposure of the HEA NPs active sites, lowering the barrier of electrons transfer in electrolysis. During the OER performance, the physical constraint effect also prevents nanoparticles from aggregating and peeling off from the LIG support under the harsh working environment, thus enhancing the durability of the catalyst. On the other hand, the carbon shell directly acts as an active site due to the electronic structure modulated by the core metal NP, which is beyond the scope of this paper. Secondly, the laser-synthesized CrMnFeCoNi HEA nanoparticles on LIG support are binder-free, which avoids the deterioration of the mass transfer and electronic conduction caused by the use of organic binder. Thirdly, the 3D interconnected porous structure of the LIG remains unchanged during the laser synthesis for enhanced electronic and mass transferring ability of the IEs.

### Extension to HEA oxide, phosphide, and sulfide nanoparticles

Taking advantage of the laser synthesis that produces a non-equilibrium state via instantaneous heating and cooling, we successfully synthesized the CrMnFeCoNi HEA oxide, phosphide, and sulfide nanoparticles. The TEM images in Fig. S13 show the oxide, phosphide, and sulfide nanoparticles have comparable size to the HEA nanoparticles. EDS elemental mapping images show the homogeneous mixing of oxygen, phosphorus, and sulfur elements in the quinary HEA elements. The incorporation of these nonmetallic atoms in HEA structure may result in severe lattice distortion, which might contribute to more exposure of metallic active sites, enhancing the electrocatalytic performance^37–39^.

In summary, we have demonstrated a laser solid-phase synthesis of high-entropy material nanoparticles by laser irradiation on a LIG substrate with a 3D porous structure. The laser-synthesized CrMnFeCoNi HEA nanoparticles on LIG support are in the hybrid form of graphene shell encapsulation. Our strategy provides simplicity, generality, and tunability to synthesize structural-uniform nanomaterials which consist of immiscible elements. The HEA/LIG is synthesized by laser-induced photothermal effect and thermionic emission, offering an active exploration in understanding the thermal decomposition process and electron emission-induced reduction of metal precursors for thermal-based techniques. Also, the CrMnFeCoNi nanoparticles loaded on LIG support can be directly used as 3D binder-free IEs, embodying the scalability of this synthesis technique. The IE-HEA/LIGs exhibit excellent electrocatalytic activity towards OER, especially the electrochemical stability. Our method is economically feasible and technically viable to synthesize composition-tunable nanomaterials, which have broad potential in energy and environmental applications.

## Materials and methods

### Sample preparation

Chromium (III) chloride hexahydrate (CrCl_3_·6H_2_O, 98%), manganese chloride (MnCl_2_, 99%), ferric (III) chloride (FeCl_3_, 99%), cobalt (II) chloride hexahydrate (CoCl_2_·6H_2_O, 99%), nickel (II) chloride hexahydrate (NiCl_2_·6H_2_O, 99.3%), ruthenium (IV) oxide (RuO_2_, >85%), polybenzimidazoles (PBI) and N, N-dimethylacetamide (DMAC) were purchased from Casmart and used as received. All chemicals were used as received without further purification.

Firstly, a homogeneous casting solution was prepared by mixing PBI (10 wt%) and DMAC (90 wt%) with continuous stirring and heating at 130 °C. Then, a thin film (40 μm in thickness) was cast on carbon paper by using a doctor blade. After that, the film was placed in deionized water vapor for 1 min before being dried under ambient conditions. After drying, the phase separation finished and the hierarchically porous PBI layers were prepared on carbon paper.

Secondly, a nanosecond laser (having a wavelength of 1064 nm, an average power of 500 W, a pulse length of 30 ns, and a laser beam diameter of 430 μm at the focusing position, JPT Opto-electronics Co., LTD., Shenzhen) was used to treat the phase-separated PBI layer. The carbonization treatment was carried out under ambient conditions with a fluence of 88 mJ/cm^2^, a 10 kHz pulse repetition rate, and a 100 mm/s scanning rate. The production is denoted by LIG using carbon paper as the substrate.

Thirdly, five metal chlorides with equal elemental molar ratios were dissolved in absolute ethanol to prepare salt solutions with concentrations of 1, 5, 10, 15, and 20 mM. Then, the LIG-coated carbon paper samples were immersed in solutions for 8 h and dried in a vacuum at 80 °C for 1 h. Then, the samples were placed in a sealed box filled with Ar gas and irradiated by the same laser as mentioned above. The laser synthesis was conducted with a variation of laser powers (15, 40, and 65 W) and fixed pulse repetition rate of 10 kHz, laser beam diameter of 430 μm, and scanning rate of 2000 mm/s. Finally, the IE with HEA nanoparticles loaded on LIG-coated carbon paper (HEA/LIG) were obtained.

### Materials characterization

Scanning electron microscopy (SEM) images were obtained by FEI Quanta 250 FEG equipped with an EDS. XRD spectrum was taken by 2D small angle X-ray scatterometer (SAXA, Xeuss 3.0 UHR) using a Cu Kα source. X-ray photoelectron spectroscopy (XPS) was taken by Kratos AXIS Supra spectrometer. Transmission electron microscopy (TEM, JEOL F200) was used to gain insight into the microstructure and elemental distribution, including dark-field (DF), high-resolution TEM (HR-TEM), SAED. All TEM samples (powders scraped off from IE-HEA/LIGs) were dispersed and sonicated in ethanol as a dilution solution and drop-casted onto the copper grid with a lacy carbon film.

### Temperature measurement and simulation

The time-dependent temperature profile of the precursor-loaded LIG under laser irradiation was recorded using an infrared camera (Multifunctional X-ray Photoelectron Spectroscope, AXIS ULTRA DLD) with a measurement range up to 2000 °C.

A two-dimensional thermal-mechanical coupled transient model was established using commercial software (ABAQUS 2018, Dassault Systemes Simulia Corp, USA). Considering the complex process and the computational efficiency, certain assumptions are made in this numerical model: (1) The laser heating source is Gaussian distribution in space; (2) The carbon paper is considered to be homogeneous and isotropic, and the laser absorptivity is regarded as a constant; (3) Effective convective heat transfer inside the molten pool was modeled without directly modeling the material flow. As shown in Fig. S9a, a rectangle is built, sized by 5.00 × 5.00 × 0.25 mm^3^ along X, Y, and Z directions. The laser beam scans along the positive X-axis with laser fluences of 58.9, 69.9 and 96.8 mJ/cm^2^, a scanning rate of 2000 mm/s, and a laser beam diameter of 0.43 mm. The coordinate values of the start and end points are (−3.5, 2.5, 0.25) and (−1.5, 2.5, 0.25), respectively. A hexahedral structure grid is employed and the localized area-related laser path is mesh-refined to improve the calculation accuracy. The physical properties of the carbon-based material are listed in Table S1.

In this model, the conduction inside the material, surface emission, and convection between the protective gas and processed material are considered following Fourier’s Law:

1 $${\rho }_{{\rm{m}}}{C}_{{\rm{m}}}\left(\frac{\partial T}{\partial t}\right)=\frac{\partial }{\partial x}\left(\lambda \frac{\partial T}{\partial x}\right)+\frac{\partial }{\partial y}\left(\lambda \frac{\partial T}{\partial y}\right)+\frac{\partial }{\partial z}\left(\lambda \frac{\partial T}{\partial z}\right)+{Q}_{{\rm{laser}}}$$

where *ρ*_m_, *C*_m_, and λ are the density, specific heat capacity, and thermal conductivity. *Q*_laser_ is the internal heating source, which can be numerically expressed as:

2 $${Q}_{{\rm{Laser}}}=\frac{2A{P}_{{\rm{Laser}}}}{\pi {r}_{0}^{2}}\exp \left(\frac{-2\left[\right.{(x-{x}_{0}-{V}_{{\rm{Laser}}}t)}^{2}+{{y}_{0}}^{2}}{{r}_{0}^{2}}\right)$$

where *A* is the laser beam absorptivity, 0.56. *P*_Laser_, *r*_0_, and *V*_Laser_ are the laser power, spot radius, and scanning rate, respectively.

The convection *Q*_c_ and surface emission *Q*_r_ can be described as:

3 $${Q}_{{\rm{c}}}=h(T-{T}_{0})$$

4 $${Q}_{{\rm{r}}}=\varepsilon k({T}^{4}-{T}_{0}^{4})$$

where *h* and *ε* are the convective coefficient and thermal emissivity, respectively. *k* is the Steffen-Boltzmann constant with a value of 5.67 × 10^−8 ^W/(m^2 ^· k^4^).

The latent heat *α*_m_ considering the phase change from solid to liquid can be expressed as:

5 $${\alpha }_{{\rm{m}}}=\frac{(1-\theta ){\rho }_{{\rm{l}}}-\theta {\rho }_{{\rm{s}}}}{2(\theta {\rho }_{{\rm{s}}}+(1-\theta ){\rho }_{{\rm{l}}})}$$

where *ρ*_s_ and *ρ*_l_ are the solid and liquid density, respectively. *θ* is the phase indicator, which can be given as:

6 $$\theta =\left\{\begin{array}{c}1\\ ({T}_{{\rm{l}}}-T)/({T}_{{\rm{l}}}-{T}_{{\rm{s}}})\\ 0\end{array}\begin{array}{c}\,T < {T}_{{\rm{s}}}\\ \,{T}_{{\rm{s}}} < {T}_{{\rm{l}}}\,\\ T > {T}_{{\rm{l}}}\end{array}\right.$$

where *T*_s_ and *T*_l_ are the solid and liquid temperatures, respectively.

### Thermionic emission measurement

The measurement set-up of thermionic emission is shown in Fig. S7a, including components of a high voltage source (Teslaman, TCM6002), precision sampling resistor (30 kΩ, 1/4 W), high-speed oscilloscope (Keysight DSO-X 3034 A) and a homemade metal-insulator structure for sample holding. The LIG samples, aluminum cathode, and anode were mounted under ambient conditions. The anode has a 10 mm diameter hole in the center and is placed 3 mm away from the cathode which is separated by a glass insulating spacer. The high voltage source can bias the anode with voltages from 0 V to 3 kV. For current measurement, the voltage on resistor R1 was measured by a differential probe and was recorded by the high-speed oscilloscope. Thanks to the ultra-low parasitic inductance of the precision resistor and wide-bandwidth differential probe, the RC constant of the current measurement system is much less than the pulse width. Therefore, the current can be directly calculated by Ohm’s law, as illustrated in Fig. S7b. Without laser illumination, the static current of both carbon paper and precursors are close to zero, which confirms the thermionic emission around 300 K can be neglected. When the nanosecond pulse laser was illuminating towards carbon paper with a 10 kHz repetition rate, a strong current signal (10 μA) could be observed. We believe this current signal was caused by the thermionic emitting of electrons from carbon paper because the measured current pulse width (3–5 μs) is orders of magnitude wider than the laser pulse (30 ns). After the substrate was changed from the carbon paper to the precursor loaded-LIG, the measured pulse current was ~5 times lower than the carbon paper and many random current peaks (cannot be aligned with laser signal) were observed in Fig. S7b. This finding indicates that the electrons induced by the laser irradiation were gained by the metal precursors which were reduced at the same time, resulting in the in-situ formation of metal atoms.

### Electrocatalytic performance

The evaluation of OER performance was carried out by a conventional three-electrode system using the VersaSTAT 4 potentiostat (AMETEK, USA). An Ag/AgCl (saturated KCl solution) electrode was used as the reference electrode, a platinum wire as the counter electrode, and the IE-HEA/LIG (0.25 cm^2^, with catalyst loading of 6.969 mg/cm^2^) as the working electrode. The measured potentials were converted to the reversible hydrogen electrode (RHE) potentials by the following equation:

7 $${{\boldsymbol{E}}}_{{\boldsymbol{RHE}}}={{\boldsymbol{E}}}_{{\boldsymbol{Ag}}/{\boldsymbol{AgCl}}}+{{\boldsymbol{E}}}_{{\boldsymbol{Ag}}/{\boldsymbol{AgCl}}}^{{\bf{0}}}+{\bf{0.059}}{\boldsymbol{pH}}$$

Where ${{\boldsymbol{E}}}_{{\boldsymbol{RHE}}}$ is the measured potential against RHE potentials, ${{\boldsymbol{E}}}_{{\boldsymbol{Ag}}{\boldsymbol{/}}{\boldsymbol{AgCl}}}$ is the measured potential against the reference electrode, and ${{\boldsymbol{E}}}_{{\boldsymbol{Ag}}{\boldsymbol{/}}{\boldsymbol{AgCl}}}^{{\boldsymbol{0}}}$ equals 0.197 V at 298 K.

The Ohmic drops within the system were compensated by applying IR-correction. The electrolyte resistance was determined by EIS at the open-circuit potential. The EIS was measured in a range of 100 kHz to 1 Hz, with a perturbation of 10 mV. The system resistance was then determined at the x-intercept of the Nyquist plot. Meantime, the Nyquist plots were recorded during OER at an overpotential of 0.5 V vs. Ag/AgCl electrode in a range of 100 kHz to 1 Hz to evaluate the catalyst/electrolyte interface characteristics.

The OER performances were tested in O_2_ saturated 1 M KOH electrolyte. The catalyst was activated using cyclic voltammetry (CV) in the range of 0 to 0.7 V vs. Ag/AgCl electrode at a scanning rate of 50 mV/s until the CV loops overlapped. The LSV curves were measured at a scanning range of 0 to 0.7 V vs Ag/AgCl. The electrodes were directly used as working electrodes.

To evaluate the OER durability of the IE, chronopotentiometric measurements at different constant current densities of 10, 20, 50, 100, and 200 mA/cm^2^ were carried out in O_2_ saturated 1.0 M KOH electrolyte.

The OER performances of purchased carbon paper and commercial RuO_2_ were measured using the same experimental setups. The purchased carbon paper can be directly used as the working electrode (0.25 cm^2^). For commercial RuO_2_, the powders (1 mg) were dispersed in a solution containing 900 μL of ethanol and 100 μL of 5 wt% Nafion, followed by sonication for 30 min. Next, 17.4 μL RuO_2_ catalyst ink was dropped onto carbon paper (0.25 cm^2^) to generate a catalyst loading of ~6.969 mg/cm^2^. After drying, the RuO_2_ catalyst-loaded carbon paper was used as the working electrodes.

## Supplementary information

Supplementary information for Supplementary Information for Laser solid-phase synthesis of graphene shell-encapsulated high-entropy alloy nanoparticles

## References

[1] Zhao, K. N. et al. High-entropy alloy nanocatalysts for electrocatalysis. *Acta Phys. Chim. Sin.* **37**, 2009077 (2021).

[2] Gao, M. C. et al. High-entropy alloys in hexagonal close-packed structure. *Metall. Mater. Trans. A* **47**, 3322–3332 (2016).

[3] Yusenko, K. V. et al. First hexagonal close packed high-entropy alloy with outstanding stability under extreme conditions and electrocatalytic activity for methanol oxidation. *Scr. Mater.* **138**, 22–27 (2017).

[4] Choi, C. et al. A highly active star decahedron Cu nanocatalyst for hydrocarbon production at low overpotentials. *Adv. Mater.* **31**, 1805405 (2019). 10.1002/adma.201805405 30549121

[5] Rekha, M. Y., Mallik, N. & Srivastava, C. First report on high entropy alloy nanoparticle decorated graphene. *Sci. Rep.* **8**, 8737 (2018). 29880871 10.1038/s41598-018-27096-8 PMC5992158

[6] Zhou, S., Jackson, G. S. & Eichhorn, B. AuPt alloy nanoparticles for CO-tolerant hydrogen activation: architectural effects in Au-Pt bimetallic nanocatalysts. *Adv. Funct. Mater.* **17**, 3099–3104 (2007).

[7] Alayoglu, S. & Eichhorn, B. Rh−Pt bimetallic catalysts: synthesis, characterization, and catalysis of core−shell, alloy, and monometallic nanoparticles. *J. Am. Chem. Soc.* **130**, 17479–17486 (2008). 19049272 10.1021/ja8061425

[8] Liu, M. M. et al. Entropy-maximized synthesis of multimetallic nanoparticle catalysts via a ultrasonication-assisted wet chemistry method under ambient conditions. *Adv. Mater. Interfaces* **6**, 1900015 (2019).

[9] Yao, Y. G. et al. Carbothermal shock synthesis of high-entropy-alloy nanoparticles. *Science* **359**, 1489–1494 (2018). 29599236 10.1126/science.aan5412

[10] Gao, S. J. et al. Synthesis of high-entropy alloy nanoparticles on supports by the fast moving bed pyrolysis. *Nat. Commun.* **11**, 2016 (2020). 32332743 10.1038/s41467-020-15934-1 PMC7181682

[11] Kim, K. S. et al. Continuous synthesis of high-entropy alloy nanoparticles by in-flight alloying of elemental metals. *Nat. Commun.* **15**, 1450 (2024). 38365786 10.1038/s41467-024-45731-z PMC10873330

[12] Ahn, J. et al. Rapid joule heating synthesis of oxide-socketed high-entropy alloy nanoparticles as CO_2_ conversion catalysts. *ACS Nano* **17**, 12188–12199 (2023). 37229643 10.1021/acsnano.3c00443

[13] Qiao, H. Y. et al. Scalable synthesis of high entropy alloy nanoparticles by microwave heating. *ACS Nano* **15**, 14928–14937 (2021). 34423972 10.1021/acsnano.1c05113

[14] Yang, G. W. Laser ablation in liquids: applications in the synthesis of nanocrystals. *Prog. Mater. Sci.* **52**, 648–698 (2007).

[15] Amendola, V. et al. Formation of alloy nanoparticles by laser ablation of Au/Fe multilayer films in liquid environment. *J. Colloid Interface Sci.* **489**, 18–27 (2017). 27770998 10.1016/j.jcis.2016.10.023

[16] Waag, F. et al. Kinetically-controlled laser-synthesis of colloidal high-entropy alloy nanoparticles. *RSC Adv.* **9**, 18547–18558 (2019). 35515245 10.1039/c9ra03254a PMC9064730

[17] Wang, B. et al. General synthesis of high-entropy alloy and ceramic nanoparticles in nanoseconds. *Nat. Synth.* **1**, 138–146 (2022).

[18] Jiang, H. Q. et al. Nanoalloy libraries from laser-induced thermionic emission reduction. *Sci. Adv.* **8**, eabm6541 (2022). 35452279 10.1126/sciadv.abm6541 PMC9032957

[19] Li, Y. et al. Laser Annealing-induced phase transformation behaviors of high entropy metal alloy, oxide, and nitride nanoparticle combinations. *Adv. Funct. Mater.* **33**, 2211279 (2023).

[20] Huang, Y. H. et al. Laser direct writing of heteroatom (N and S)-doped graphene from a polybenzimidazole ink donor on polyethylene terephthalate polymer and glass substrates. *Small* **14**, 1803143 (2018). 10.1002/smll.201803143 30284372

[21] Sharma, M. et al. Work function-tailored graphene *via* transition metal encapsulation as a highly active and durable catalyst for the oxygen reduction reaction. *Energy Environ. Sci.* **12**, 2200–2211 (2019).

[22] Yoo, J. M. et al. Carbon shell on active nanocatalyst for stable electrocatalysis. *Acc. Chem. Res.* **55**, 1278–1289 (2022). 35436084 10.1021/acs.accounts.1c00727

[23] Moreno-Castilla, C. Adsorption of organic molecules from aqueous solutions on carbon materials. *Carbon* **42**, 83–94 (2004).

[24] Li, H. B. et al. Mechanisms of metal sorption by biochars: biochar characteristics and modifications. *Chemosphere* **178**, 466–478 (2017). 28342995 10.1016/j.chemosphere.2017.03.072

[25] Yang, X. D. et al. Surface functional groups of carbon-based adsorbents and their roles in the removal of heavy metals from aqueous solutions: a critical review. *Chem. Eng. J.* **366**, 608–621 (2019). 34522159 10.1016/j.cej.2019.02.119 PMC8437042

[26] Eustathopoulos, N., Nicholas, M. G. & Drevet, B. Wettability at High Temperatures. (Amsterdam: Pergamon, 1999).

[27] Sha, Y. et al. 3D binder-free integrated electrodes prepared by phase separation and laser induction (PSLI) method for oxygen electrocatalysis and zinc–air battery. *Adv. Energy Mater.* **12**, 2200906 (2022).

[28] Zhang, T. F. et al. Macroscopic and direct light propulsion of bulk graphene material. *Nat. Photonics* **9**, 471–476 (2015).

[29] Wei, X. L. et al. Breakdown of Richardson’s law in electron emission from individual self-joule-heated carbon nanotubes. *Sci. Rep.* **4**, 5102 (2014). 24869719 10.1038/srep05102 PMC4037708

[30] Qiu, H. J. et al. Noble metal-free nanoporous high-entropy alloys as highly efficient electrocatalysts for oxygen evolution reaction. *ACS Mater. Lett.* **1**, 526–533 (2019).

[31] Zhang, G. L. et al. High entropy alloy as a highly active and stable electrocatalyst for hydrogen evolution reaction. *Electrochim. Acta* **279**, 19–23 (2018).

[32] He, B. B., Zu, Y. & Mei, Y. Design of advanced electrocatalysts for the high-entropy alloys: principle, progress, and perspective. *J. Alloy. Compd.* **958**, 170479 (2023).

[33] Chandrasekaran, S. et al. Developments and perspectives on robust nano- and microstructured binder-free electrodes for bifunctional water electrolysis and beyond. *Adv. Energy Mater.* **12**, 2200409 (2022).

[34] Yan, X. X., Ha, Y. & Wu, R. B. Binder-free air electrodes for rechargeable zinc-air batteries: recent progress and future perspectives. *Small Methods* **5**, 2000827 (2021). 10.1002/smtd.202000827 34927848

[35] Yan, Z. X. et al. Graphene nanosphere as advanced electrode material to promote high performance symmetrical supercapacitor. *Small* **17**, 2007915 (2021). 10.1002/smll.202007915 33749142

[36] Huang, Z. N. et al. Direct observation of the formation and stabilization of metallic nanoparticles on carbon supports. *Nat. Commun.* **11**, 6373 (2020). 33311508 10.1038/s41467-020-20084-5 PMC7733500

[37] Zhang, L. J., Cai, W. W. & Bao, N. Z. Top-level design strategy to construct an advanced high-entropy Co–Cu–Fe–Mo (Oxy)hydroxide electrocatalyst for the oxygen evolution reaction. *Adv. Mater.* **33**, 2100745 (2021). 10.1002/adma.202100745 33876867

[38] Cui, M. J. et al. High-entropy metal sulfide nanoparticles promise high-performance oxygen evolution reaction. *Adv. Energy Mater.* **11**, 2002887 (2021).

[39] Liu, K. W. et al. High-performance transition metal phosphide alloy catalyst for oxygen evolution reaction. *ACS Nano* **12**, 158–167 (2018). 29211437 10.1021/acsnano.7b04646

