# Supplementary material for: Laser solid-phase synthesis of graphene shell-encapsulated high-entropy alloy nanoparticles
Source: Light Sci Appl. 2024 Sep 26;13:270. doi: 10.1038/s41377-024-01614-y (PMC11427457; doi:10.1038/s41377-024-01614-y)
Supplement: Supplementary file 1 — Supplementary information for Supplementary Information for Laser solid-phase synthesis of graphene shell-encapsulated high-entropy alloy nanoparticles [file 41377_2024_1614_MOESM1_ESM.docx]

**Supplementary Information for**

**Laser solid-phase synthesis of graphene shell-encapsulated high-entropy alloy nanoparticles**

Yuxiang Liu, Jianghuai Yuan, Jiantao Zhou, Kewen Pan, Ran Zhang, Rongxia Zhao, Lin Li, Yihe Huang*, Zhu Liu*

Research Centre for Laser Extreme Manufacturing,

Ningbo Institute of Materials Technology and Engineering,

Chinese Academy of Sciences, Ningbo 315201, China


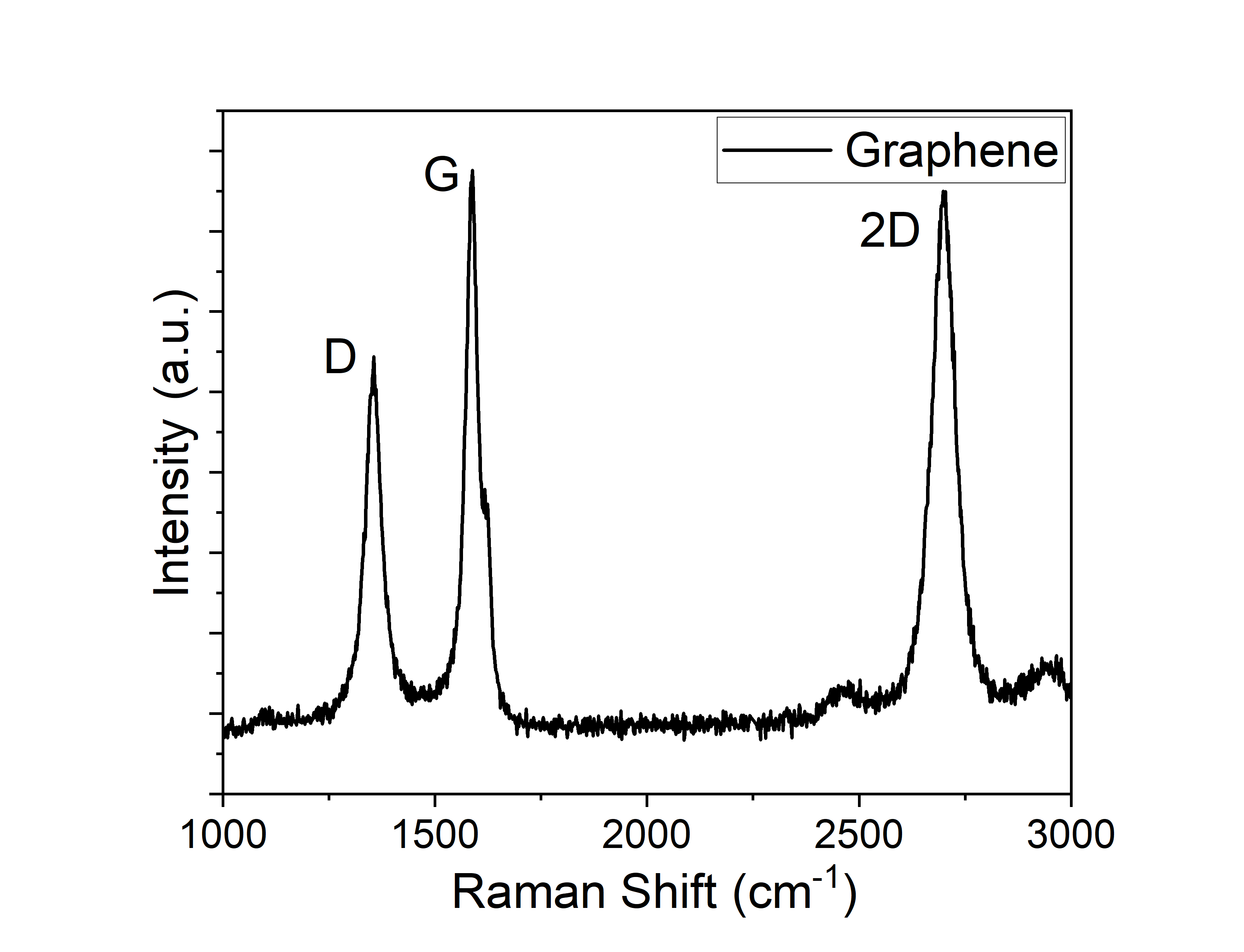
**Fig. S1** Raman spectrum of the laser-induced graphene (LIG) containing defects.


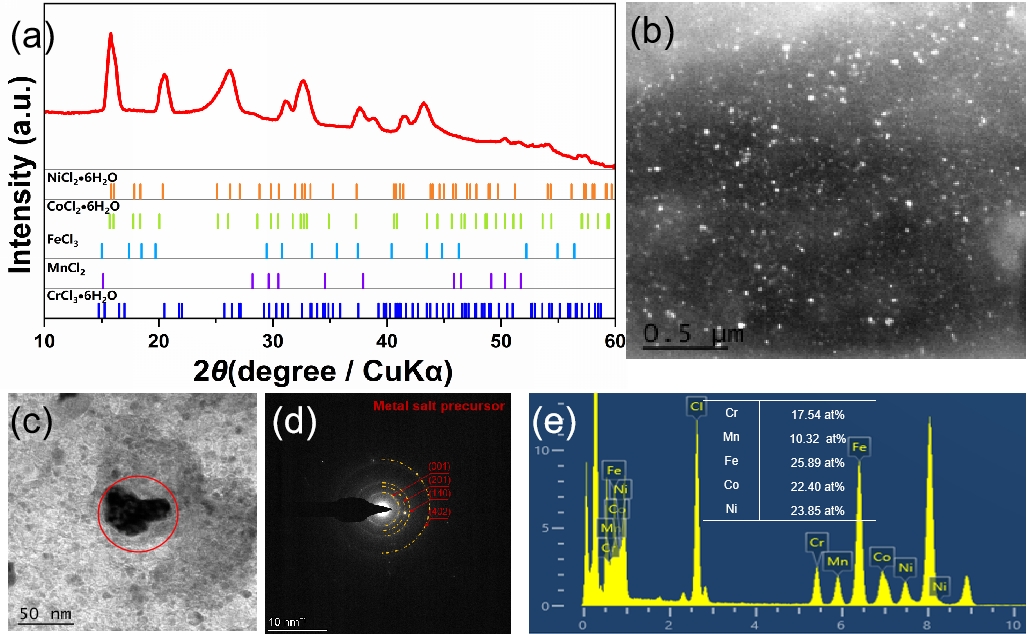


**Fig. S2** Characterization of the mixed metal precursors. (a) XRD pattern (0.01 M), (b-e) TEM images, SAED pattern, and elemental compositions.


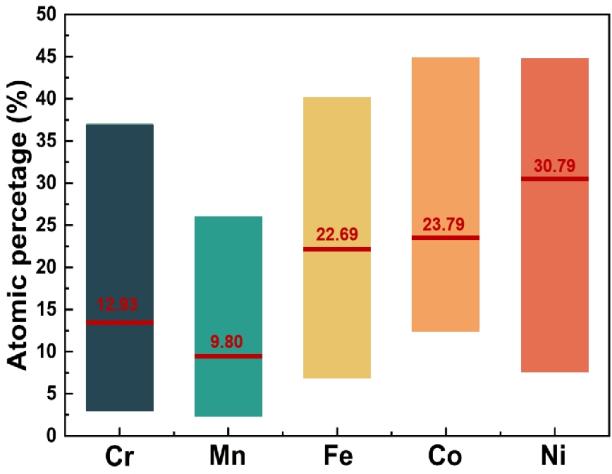


**Fig. S3** Elemental ratios of the CrMnFeCoNi HEA nanoparticles obtained from EDS measurements.


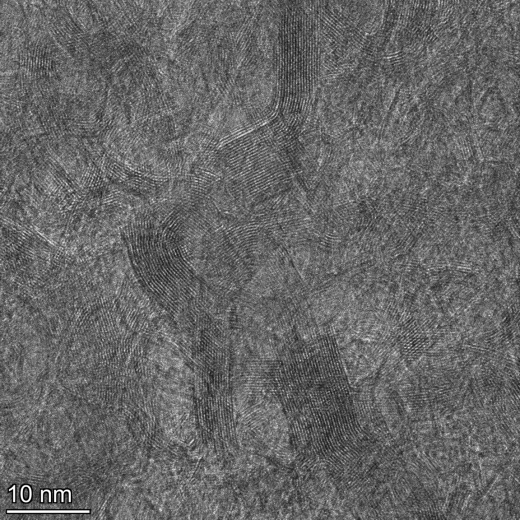

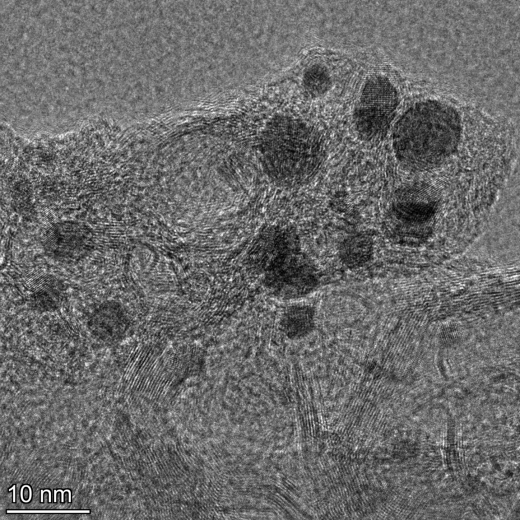

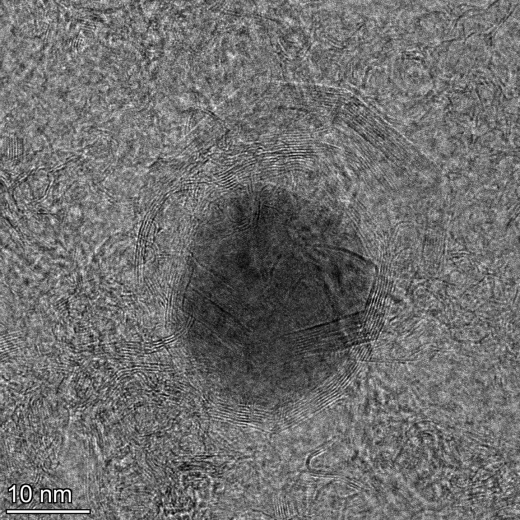

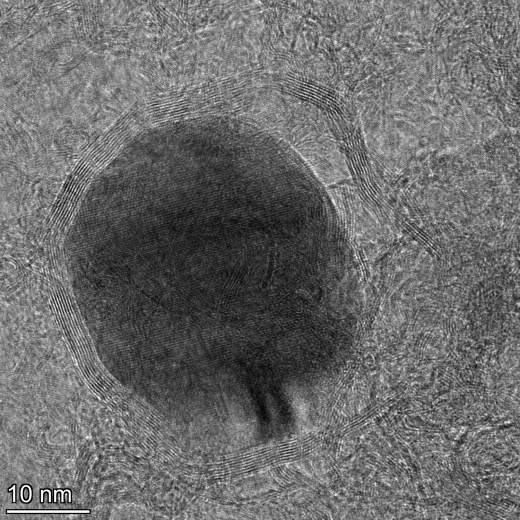


(d)

(c)

(b)

(a)

**Fig. S4** HRTEM images of (a) LIG and (b-d) CrMnFeCoNi HEA nanoparticles. Note: the nanoparticles are embraced by several graphene layers.


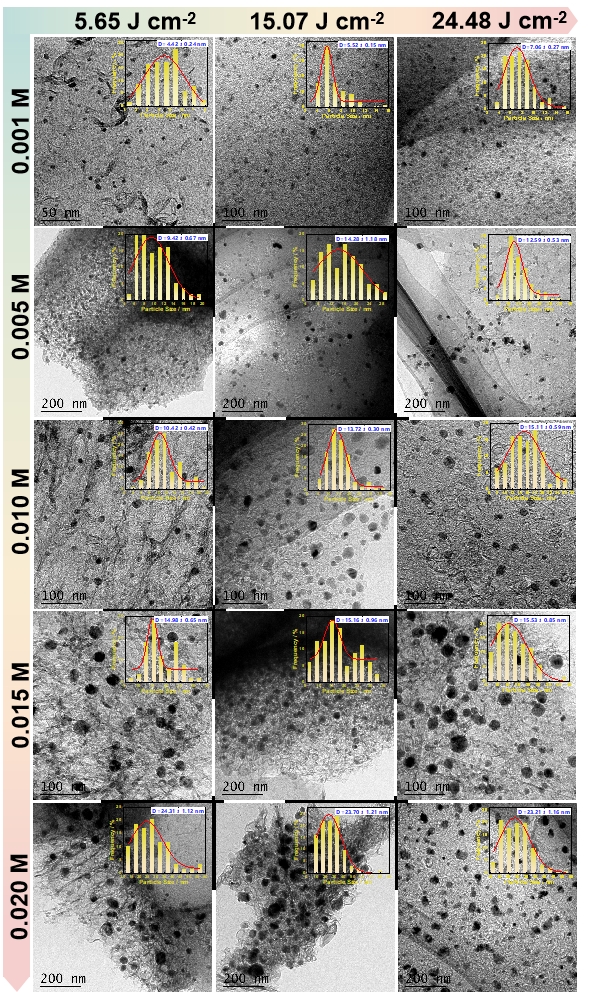


**Fig.S5** TEM images and particle size distributions of CrMnFeCoNi HEA/LIG at different laser fluences (58.9, 69.9 and 96.8 mJ cm^-2^) and precursor concentrations (0.001, 0.005, 0.01, 0.015 and 0.02 M).
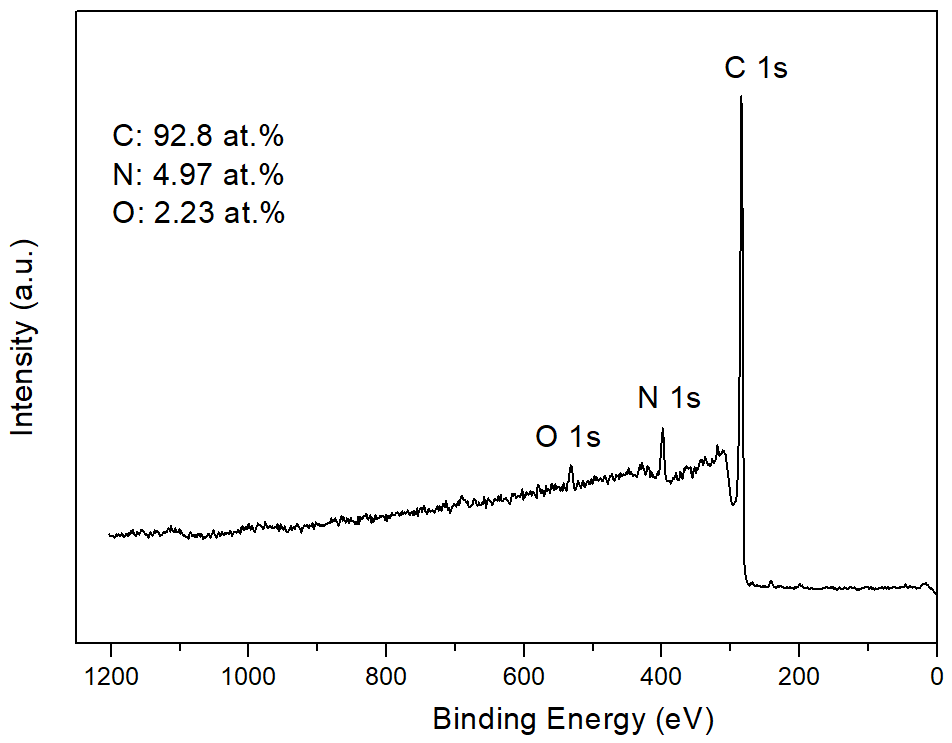

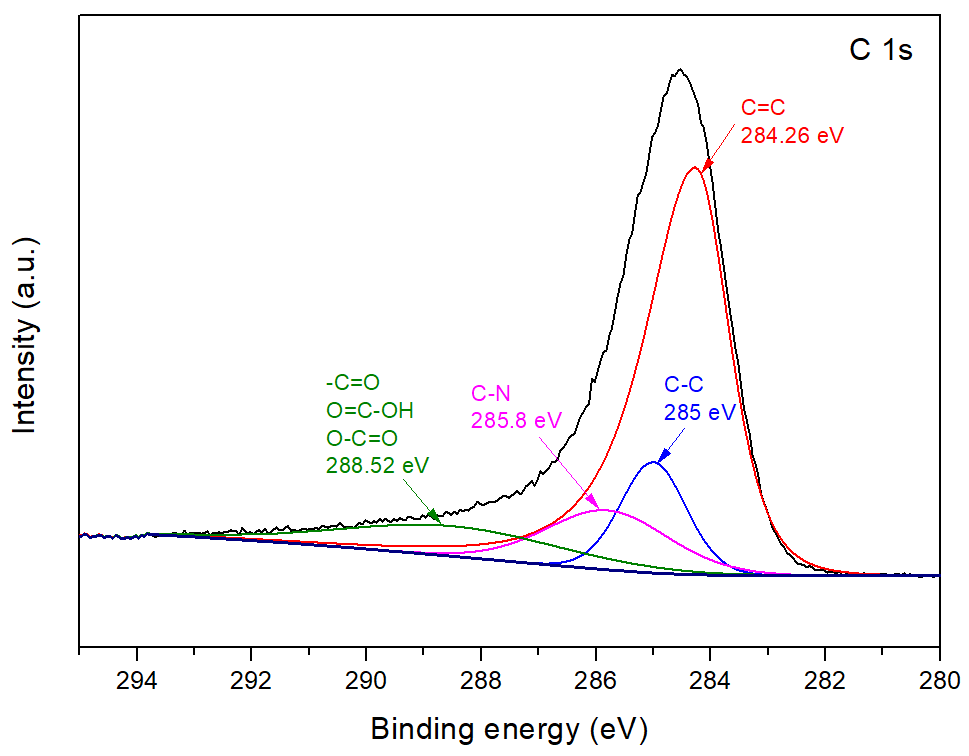


(b)

(a)


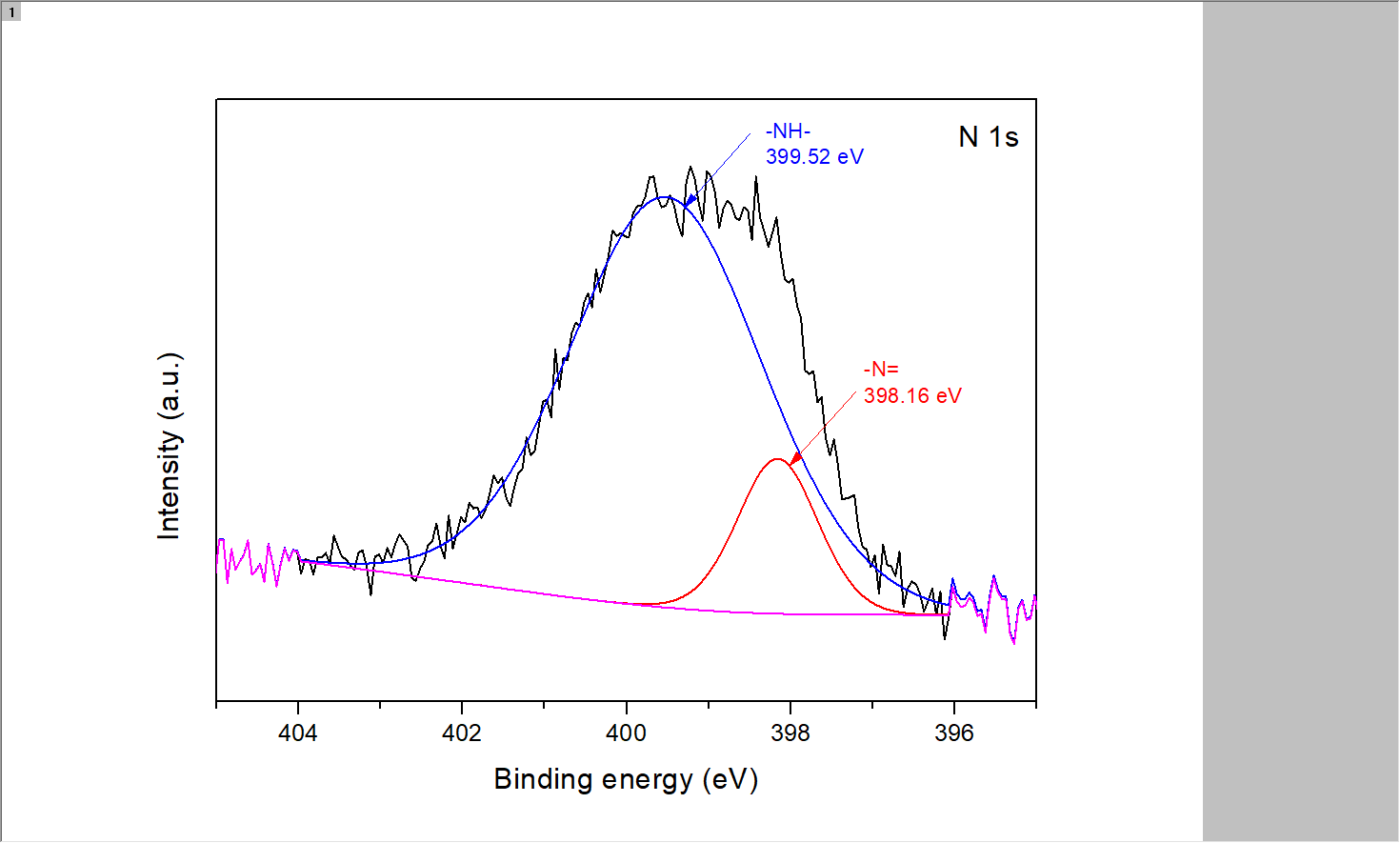

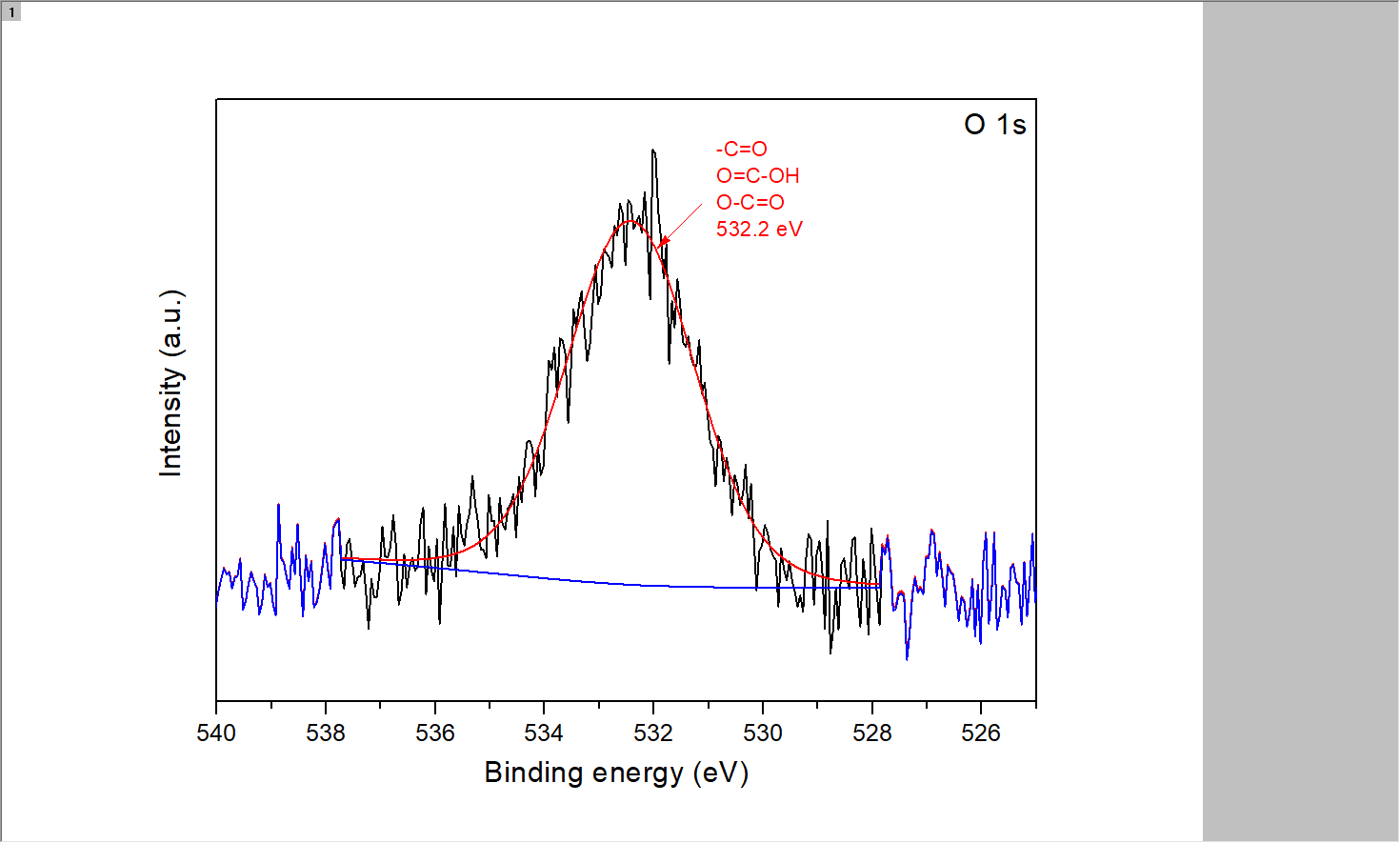


(d)

(c)

**Fig. S6** XPS spectra. (a) Wide spectra of the LIG, (b−d) Characteristic peaks of C 1s, N 1s, and O 1s, respectively.


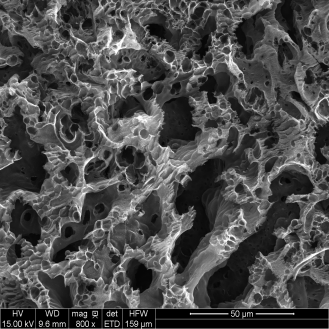

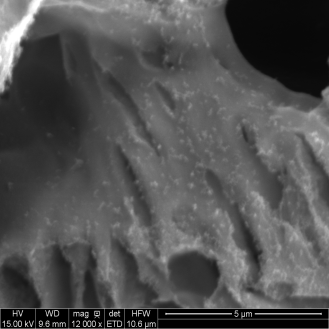

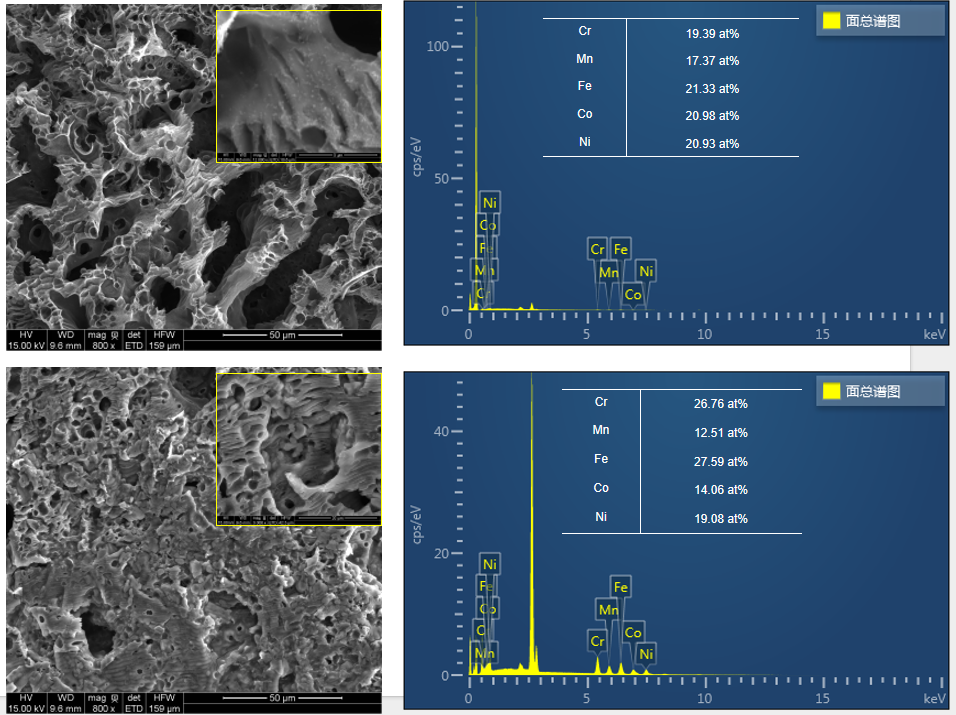

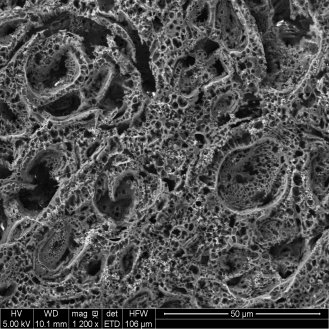

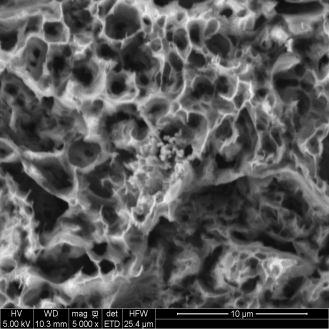

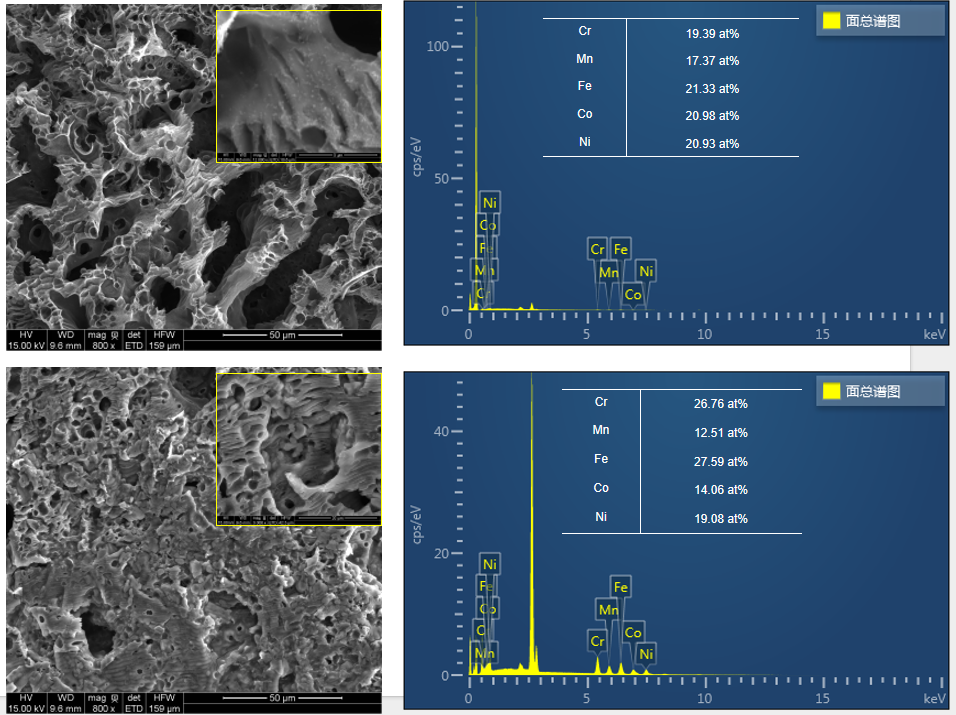


(b.1)

(c)

(b)

(d)

(d.1)

(a)

**Fig. S7** SEM images of the metal precursor loaded LIG at the precursor concentration of 0.005 M (a, b) and 0.02 M (c,d), and the corresponding elemental distribution for the SEM images of c and d.

**
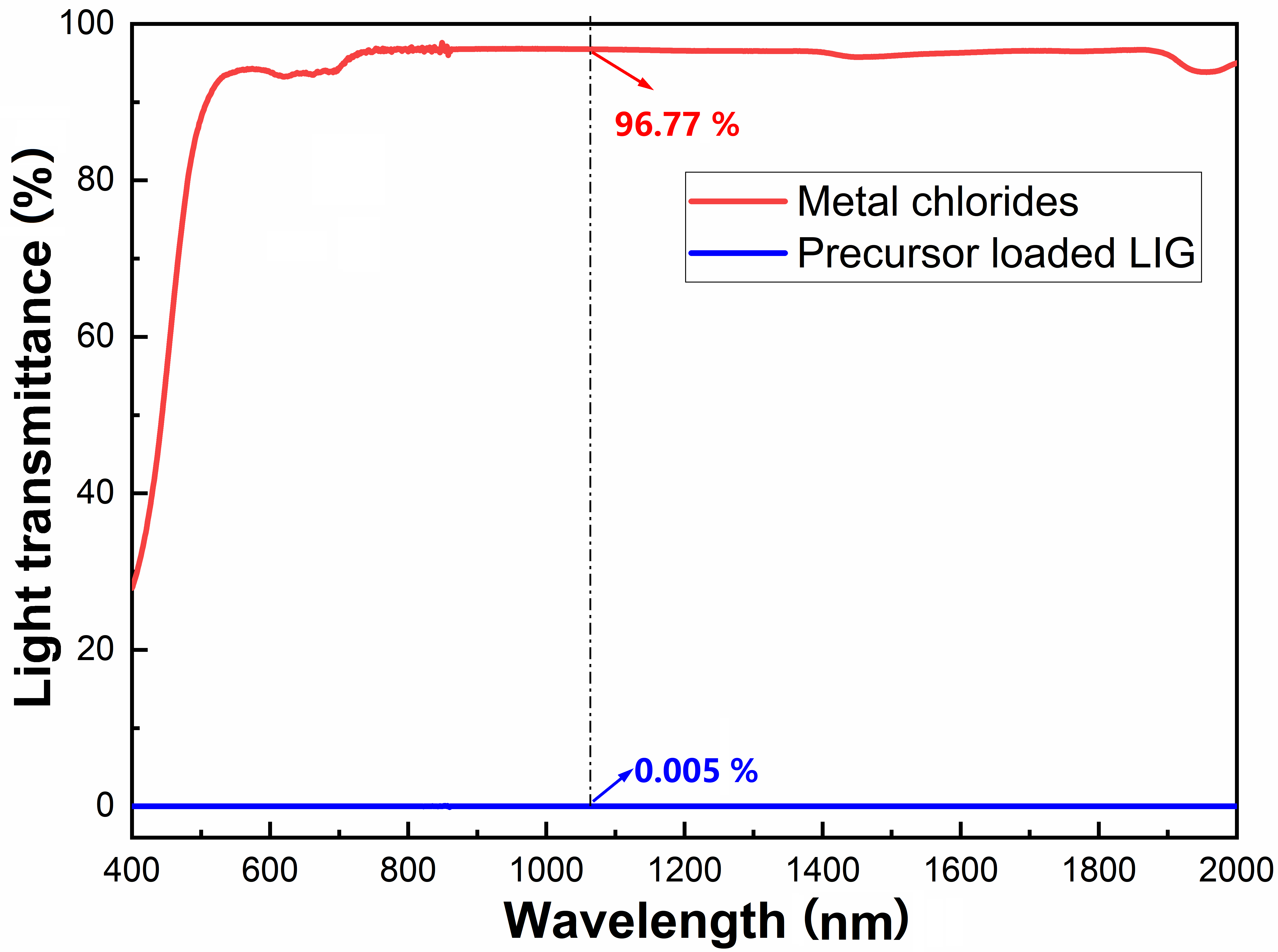
**

**Fig. S8** UV-Vis transmittance of the mixed metal chlorides on silica glass and the precursor-loaded LIG.


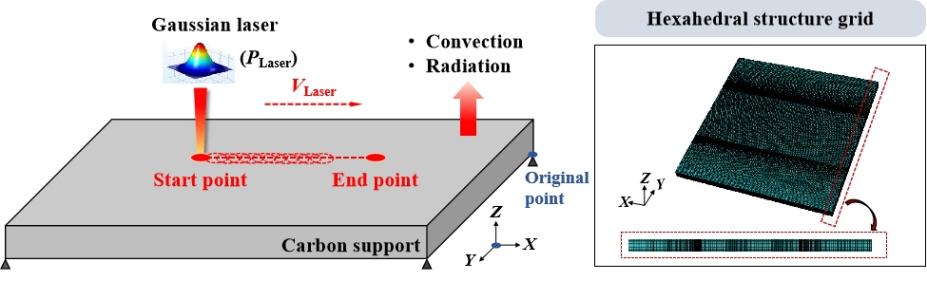

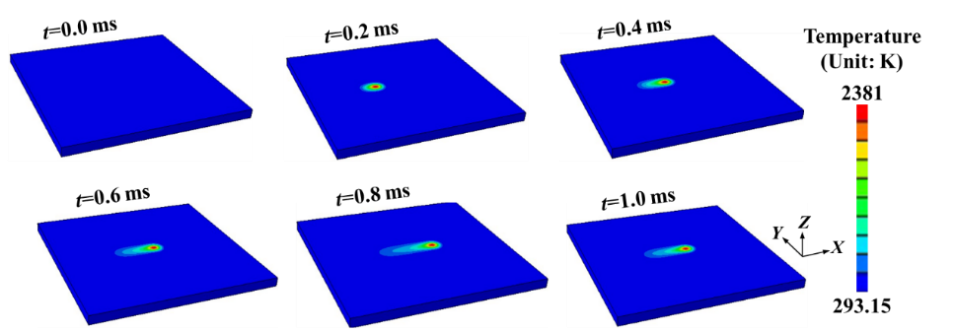

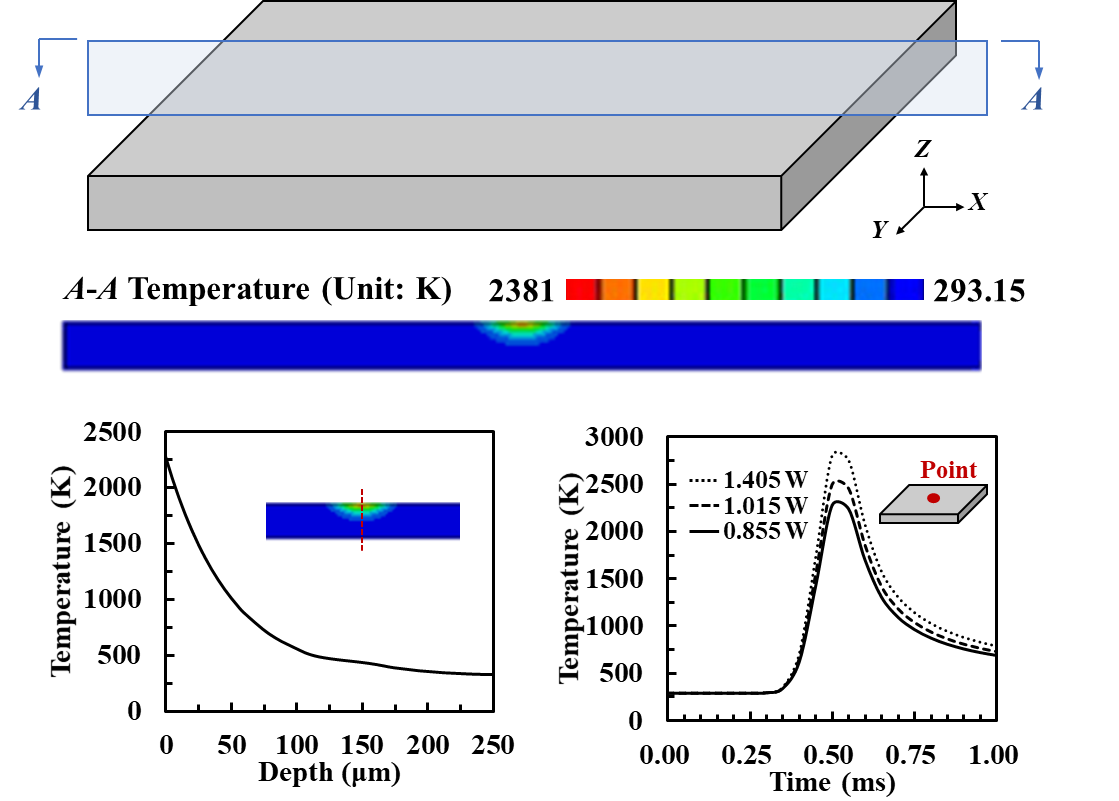


(c)

(a)

(b)

**Fig. S9** Simulation results. (a) Simulation model of laser irradiation of carbon-based support. (b) Temperature distribution during laser irradiation of carbon support. (c) Symmetrical temperature profile.


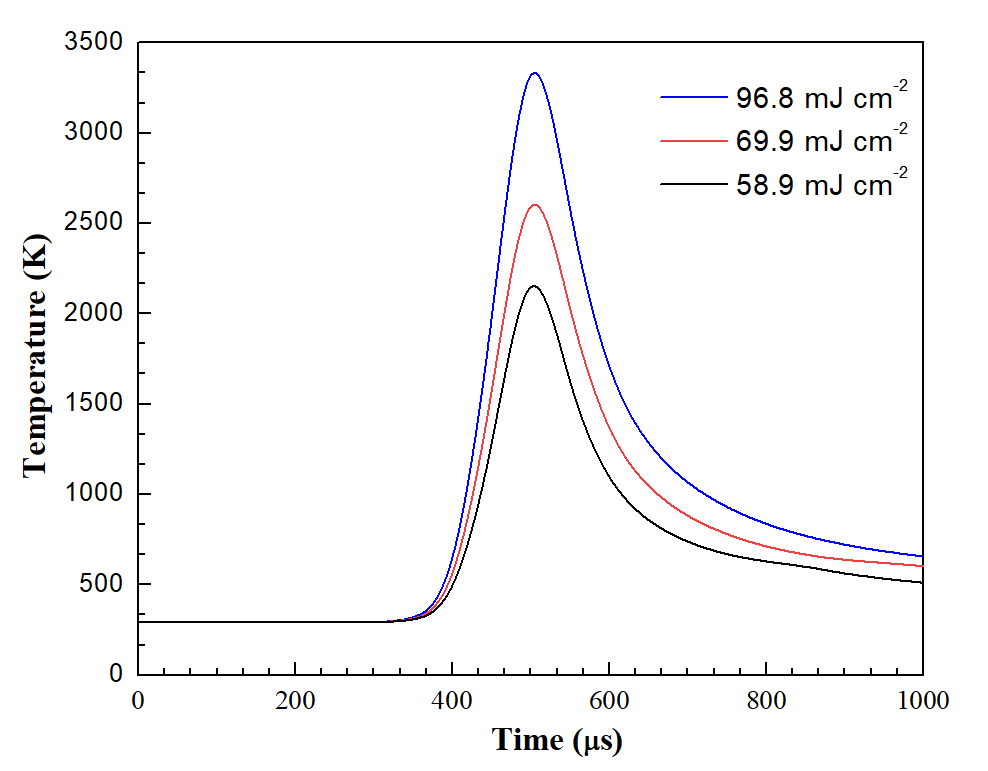

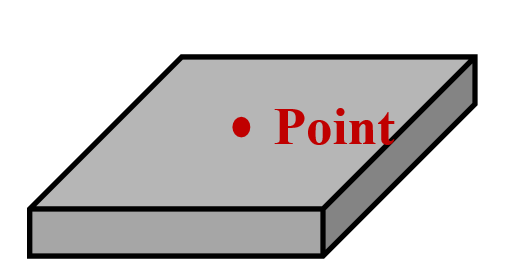

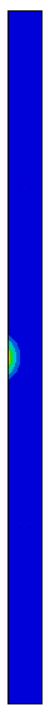

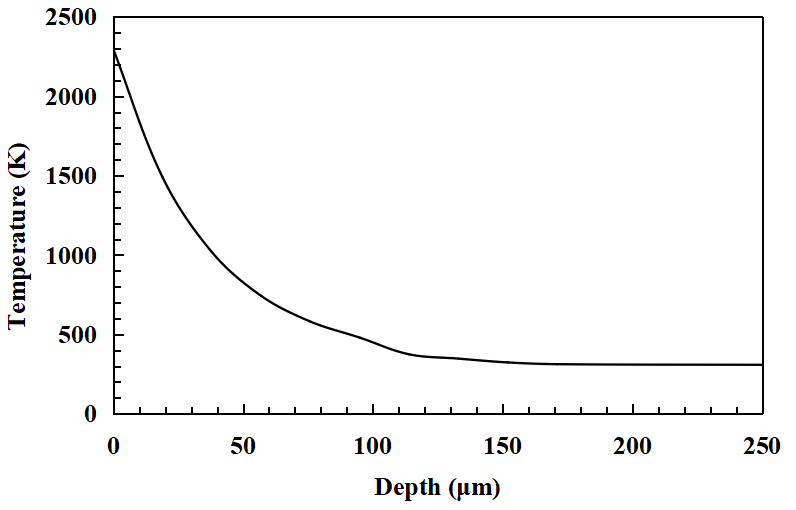


(b)

(a)

**Fig. S10** Simulation results. (a) Transient thermal history for the centre point on the upper surface at different laser fluences. (b) Temperature gradient at the selected point along the negative Z-axis.


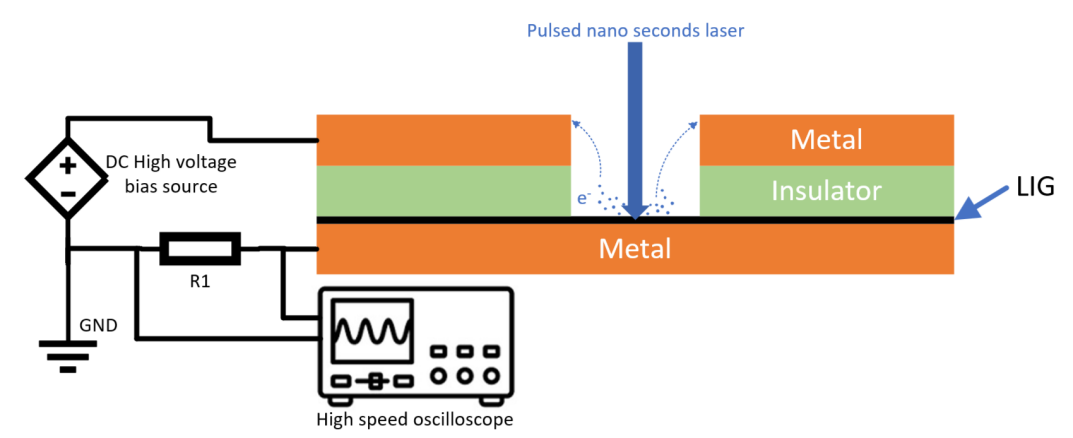


(a)


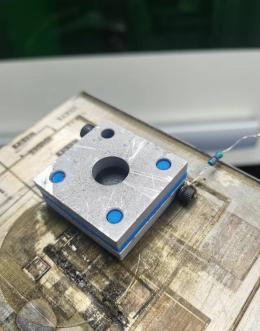

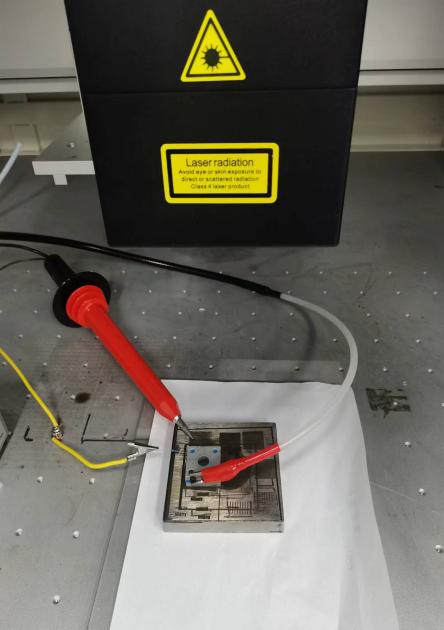
 **
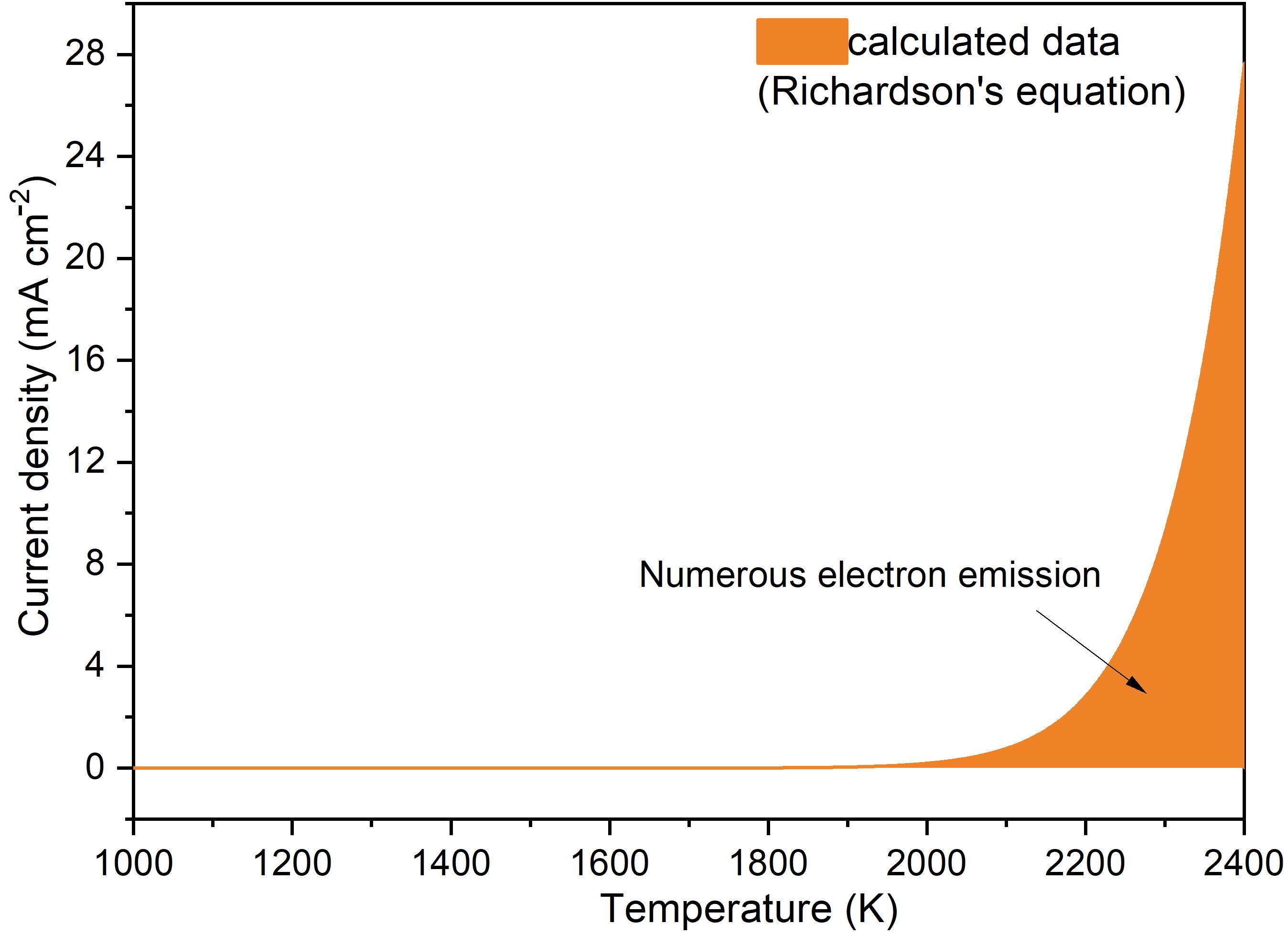
**

(c)

(b)

**Fig. S11** Laser-induced thermionic emission measurements. (a) Measurement setup. (b) Custom-built cell to measure the laser-induced current flow. (c) Calculated emission current density as a function of local temperatures according to Richardson's equation.


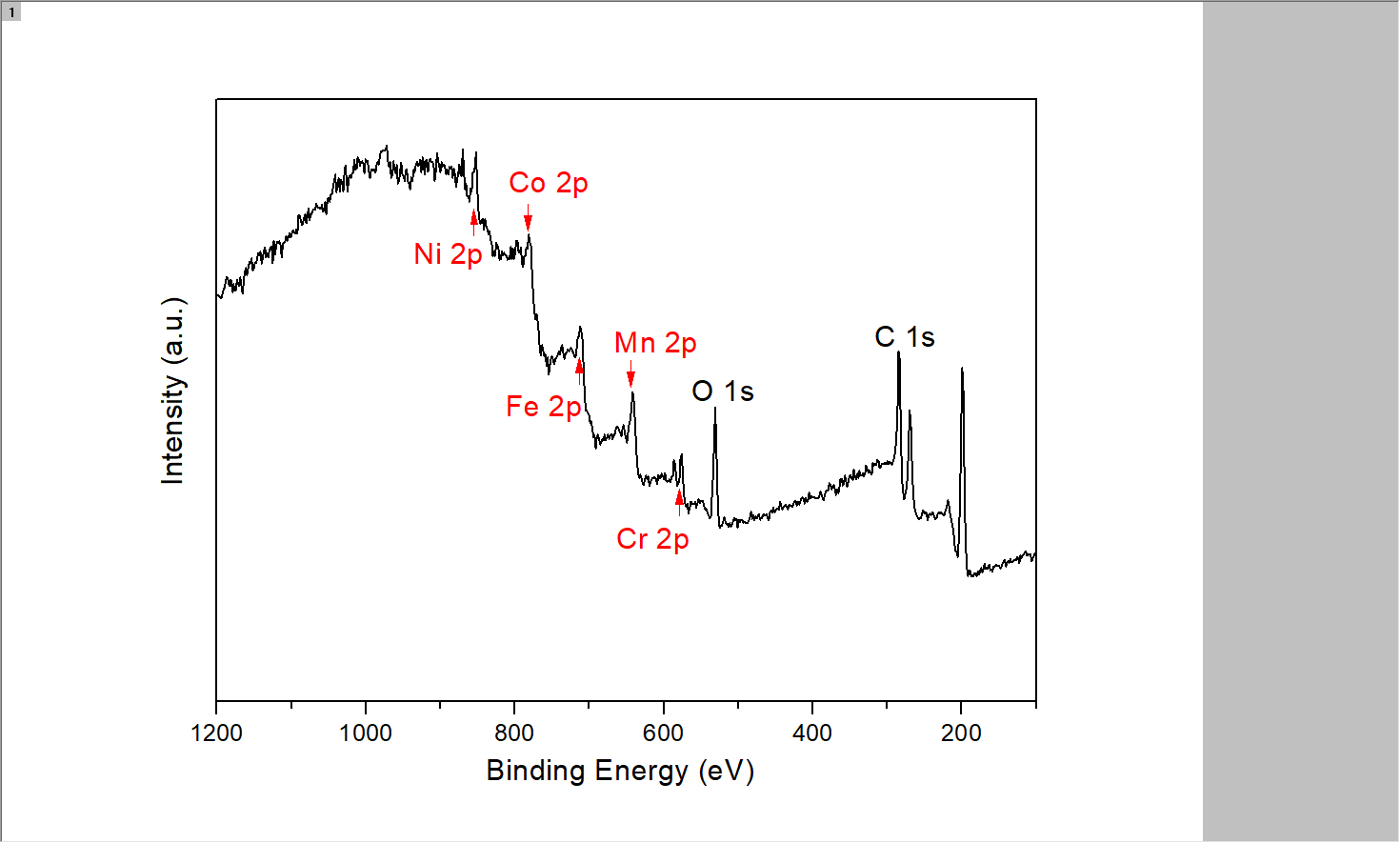

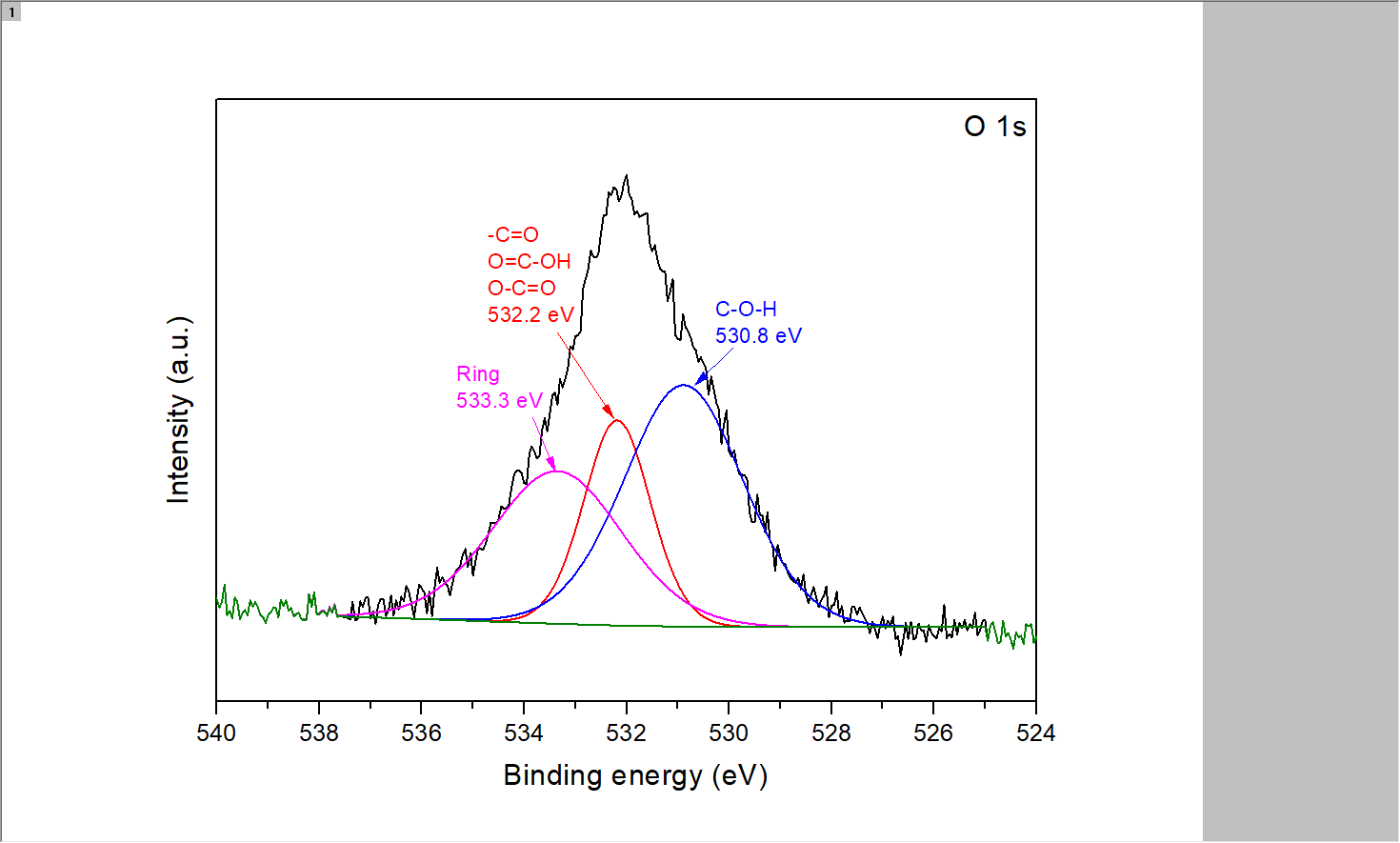

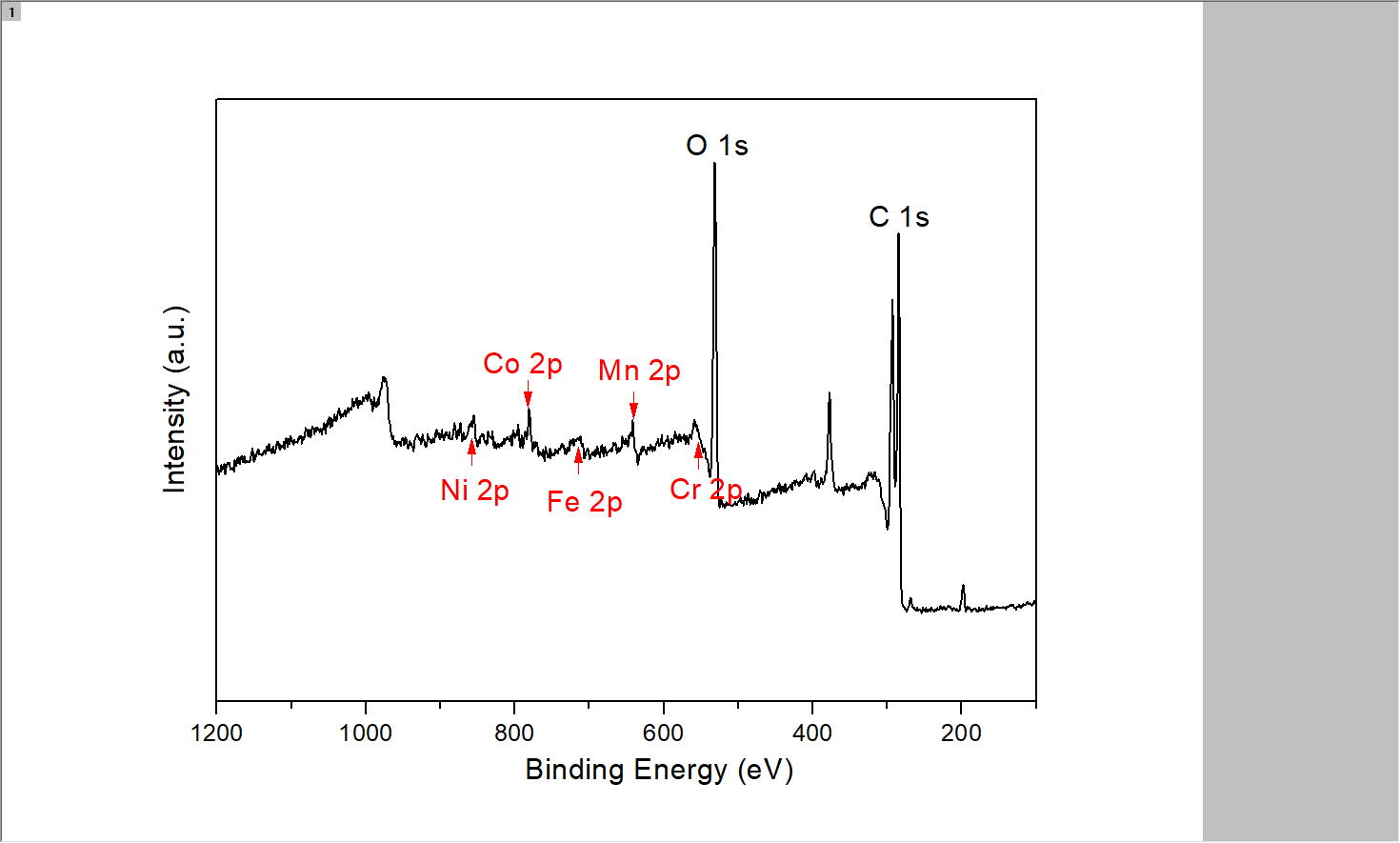

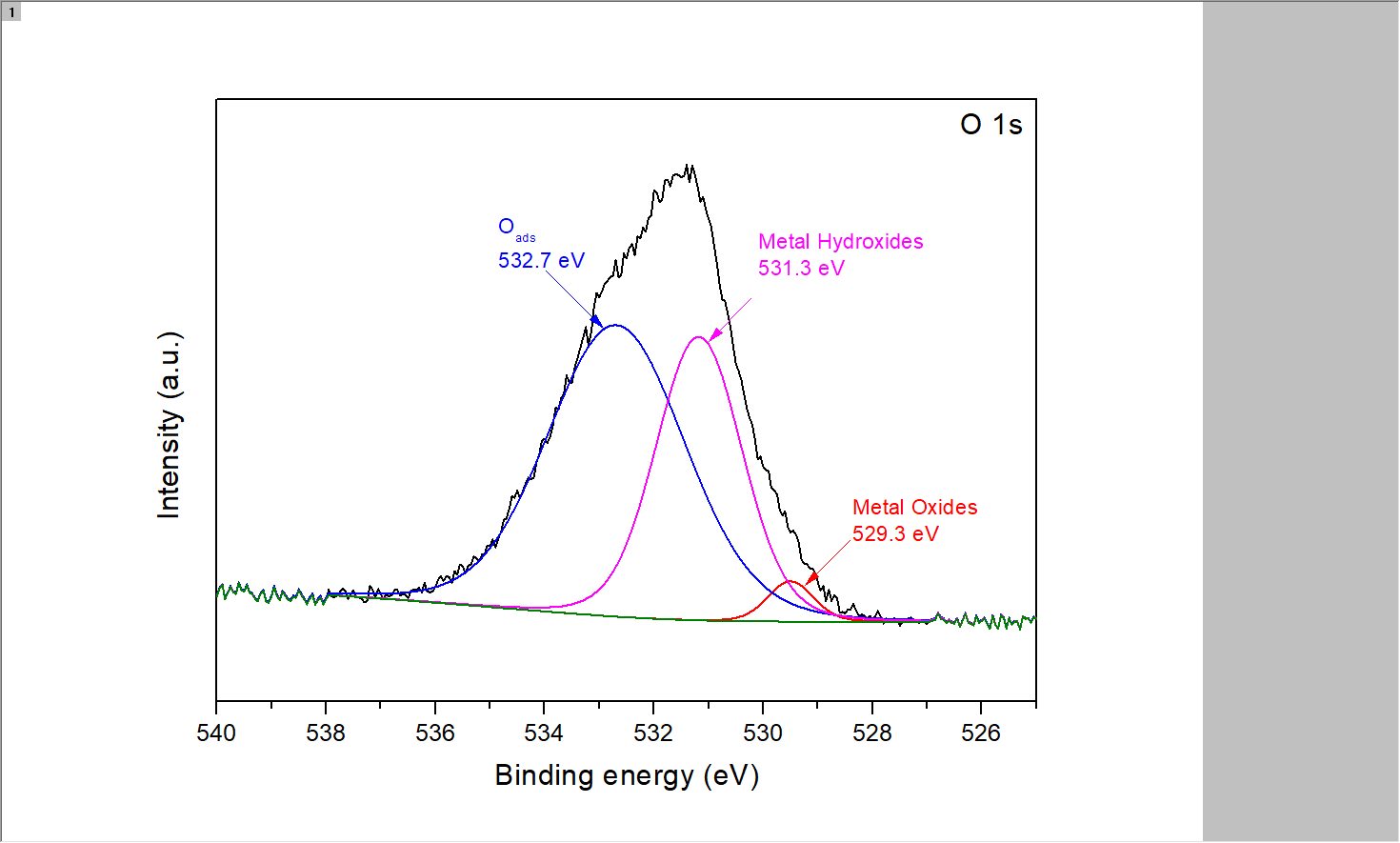


(a)

(b)

(d)

(c)

**Fig. S12** (a,b) XPS wide spectra of HEA/LIG and characteristic peaks of O 1s. (c-d) XPS wide spectra of IE-HEA/LIG and characteristic peaks of O 1s after OER tests.

**
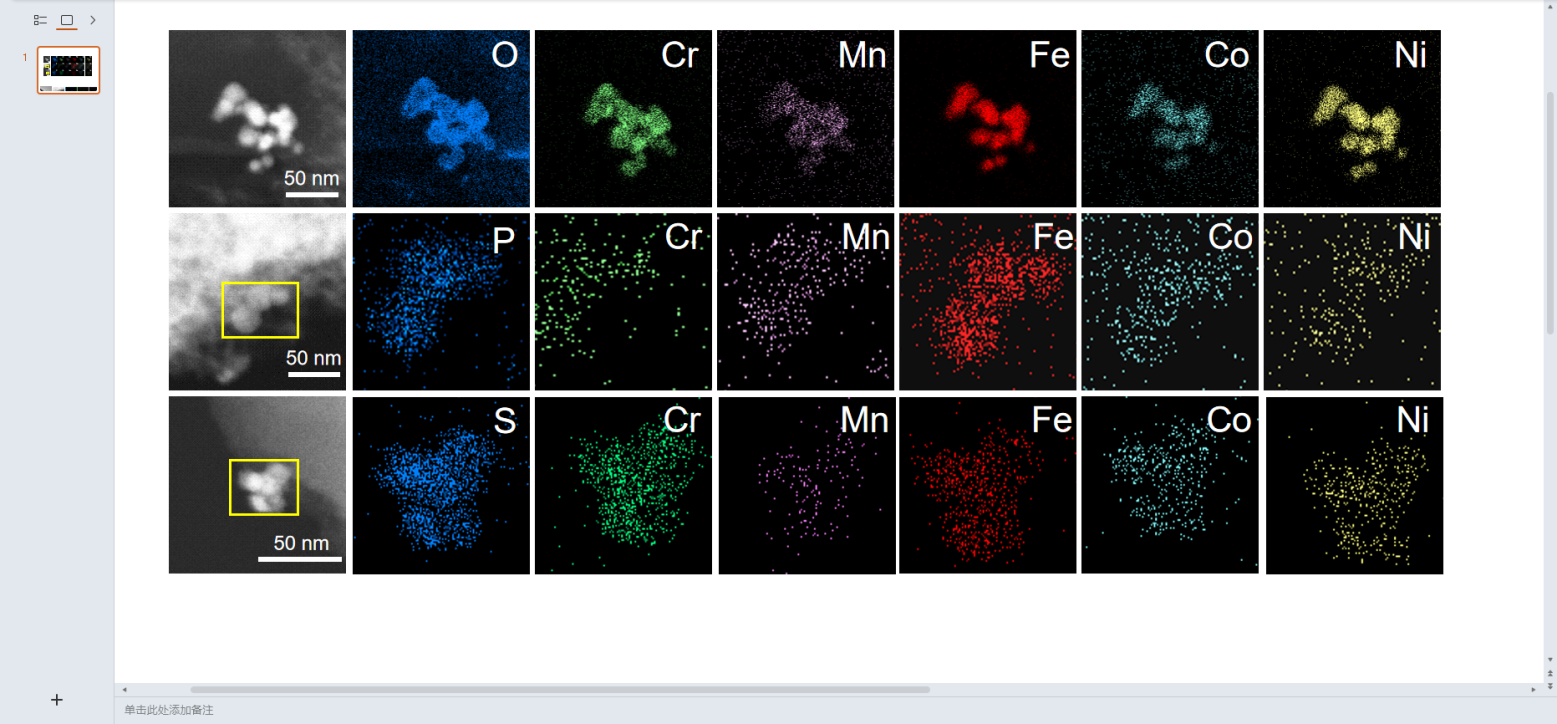
**

(b)

(c)

(a)

**Fig. S13** TEM images and elemental mappings of (a) HEA-O, (b) HEA-P, and (c) HEA-S NPs on LIG.

**Table S1** Material properties in the temperature simulation [1-3].

| Property (unit) | Symbol | Value |
| --- | --- | --- |
| Density (kg m^-3^) | ρ_s_ | 1780 |
| Boiling point (K) | T_v_ | 3650 |
| Latent heat (J kg^-1^) | L | 5.03 × 10^5^ |
| Thermal conductivity (W m^-1^ K^-1^) | λ | 50 |
| Specific heat capacity (J kg^-1^ K^-1^) | C_P_ | 875 |
| Emission | ɛ | 0.14 |
| Absorptivity | A | 0.56 |
| Convection efficient (W m^2^ K) | h_c_ | 20 |

**Table S2** Pyrolysis temperature and chemical reduction potential of the precursors used in this study.

| Metal chlorides | Pyrolysis temperature (K) | Chemical reduction potential (V) |
| --- | --- | --- |
| CrCl_3_ | ＞ 1573 | -0.407 |
| MnCl_2_ | ＞ 773 | -1.185 |
| FeCl_3_ | ＞ 588 | -0.44 |
| CoCl_2_ | 673-773 | -0.28 |
| NiCl_2_ | ＞ 1246 | -0.25 |

**Table S3** Physical properties of Cr, Mn, Fe, Co, Ni.

| Metal | Melting point (K) | Boiling point (K) | Electron configuration | Electro- negativity | Atomic radium (pm) |
| --- | --- | --- | --- | --- | --- |
| Cr | 2180 | 2944 | [Ar]3d^5^4s^1^ | 1.66 | 128 |
| Mn | 1519 | 2334 | [Ar]3d^5^4s^2^ | 1.55 | 127 |
| Fe | 1808 | 3343 | [Ar]3d^6^4s^2^ | 1.83 | 126 |
| Co | 1768 | 3200 | [Ar]3d^7^4s^2^ | 1.88 | 125 |
| Ni | 1728 | 3186 | [Ar]3d^8^4s^2^  [Ar]3d^9^4s^1^ | 1.91 | 124 |

**Table S4** Comparison of OER performance with the most recently reported HEA catalysts in 1 M KOH.

| Catalysts | Electrolytes | Overpotential (mV)  @ 10 mA cm^-2^ | Tafel slope  (mV dec^-1^) | Stability  (h) | Refs. |
| --- | --- | --- | --- | --- | --- |
| CoCrFeNiMo | 1 M KOH | 220 | 59 | 24 | 4 |
| FeCoNiCrMn | 1 M KOH | 345  @50 mA cm^-2^ | 81.3 | 20 | 5 |
| FeCoNiMnCu | 1 M KOH | 280 | 59 | 40 | 6 |
| MnFeCoNiCu | 1 M KOH | 263 | 43 | 24 | 7 |
| AlNiCoFeMo | 1 M KOH | 240 | 46 | 50 | 8 |
| AlFeCoNiCr | 1 M KOH | 240 | 52 | / | 9 |
| AlCrCuFeNi | 1 M KOH | 270 | 77.5 | 35 | 10 |
| FeNiMnCrCu | 1 M KOH | 314 | 58 | 10 | 11 |
| MnFeCoNiCu | 1 M KOH | 245 | 54 | 48 | 12 |
| CoFeGaNiZn | 1 M KOH | 370 | 71 | 10 | 13 |
| CoCuFeNiMnMo | 1 M KOH | 375 | 125 | 72 | 14 |
| FeCoNiMoW | 1 M KOH | 233 | 36.7 | 115 | 15 |
| CrMnFeCoNi | 1 M KOH | 265 | 37.9 | 30 | 16 |
| NiFeCoCuMn | 1 M KOH | 226 | 58.2 | 10 | 17 |
| FeCoNiMnCr | 1 M KOH | 228 | 45.39 | 48 | 18 |
| NiFeCuCoCe | 1 M KOH | 219 | 110 | 100 | 19 |
| CrMnFeCoNi | 1 M KOH | 250 | 41.5 | 24 | 20 |
| AlNiCoRuMo | 1 M KOH | 245 | 54.5 | 100 | 21 |
| FeCoNiCuPtIr | 1 M KOH | 255 | 61.7 | 20 | 22 |
| FeCoNiRu | 1 M KOH | 243 | 45 | 40 | 23 |
| Pt_34_Fe_5_Ni_20_Cu_31_Mo_9_Ru | 1 M KOH | 259 | 39 | 40 | 24 |
| CrMnFeCoNi | 1 M KOH | 293 | 57.9 | **428** | This work |

**References**

1. Nakamiya, T.; Ueda, T.; Ikegami, T.; Mitsugi, F.; Ebihara, K.; Sonoda, Y.; Iwasaki, Y.; Tsuda, R., Effect of a pulsed Nd:YAG laser irradiation on multi-walled carbon nanotubes film. *Thin Solid Films* **2009,** *517* (14), 3854-3858.

2. Keuntje, J.; Mrzljak, S.; Gerdes, L.; Wippo, V.; Kaierle, S.; Walther, F.; Jaeschke, P., Finite Element Simulation and Experimental Assessment of Laser Cutting Unidirectional CFRP at Cutting Angles of 45° and 90°. *Polymers* **2023,** *15* (18), 3851.

3. Deng, L.; Young, R. J.; Kinloch, I. A.; Sun, R.; Zhang, G.; Noé, L.; Monthioux, M., Coefficient of thermal expansion of carbon nanotubes measured by Raman spectroscopy. *Applied Physics Letters* **2014,** *104* (5), 051907.

4. Tang, J.; Xu, J. L.; Ye, Z. G.; Li, X. B.; Luo, J. M., Microwave sintered porous CoCrFeNiMo high entropy alloy as an efficient electrocatalyst for alkaline oxygen evolution reaction. *Journal of Materials Science & Technology* **2021,** *79*, 171-177.

5. Li, H.; Zhu, H.; Sun, S.; Hao, J.; Zhu, Z.; Xu, F.; Lu, S.; Duan, F.; Du, M., Thermodynamically driven metal diffusion strategy for controlled synthesis of high-entropy alloy electrocatalysts. *Chemical Communications* **2021,** *57* (78), 10027-10030.

6. Huang, K.; Peng, D.; Yao, Z.; Xia, J.; Zhang, B.; Liu, H.; Chen, Z.; Wu, F.; Wu, J.; Huang, Y., Cathodic plasma driven self-assembly of HEAs dendrites by pure single FCC FeCoNiMnCu nanoparticles as high efficient electrocatalysts for OER. *Chemical Engineering Journal* **2021,** *425*, 131533.

7. Huang, K.; Zhang, B.; Wu, J.; Zhang, T.; Peng, D.; Cao, X.; Zhang, Z.; Li, Z.; Huang, Y., Exploring the impact of atomic lattice deformation on oxygen evolution reactions based on a sub-5 nm pure face-centred cubic high-entropy alloy electrocatalyst. *Journal of Materials Chemistry A* **2020,** *8* (24), 11938-11947.

8. Qiu, H.-J.; Fang, G.; Gao, J.; Wen, Y.; Lv, J.; Li, H.; Xie, G.; Liu, X.; Sun, S., Noble Metal-Free Nanoporous High-Entropy Alloys as Highly Efficient Electrocatalysts for Oxygen Evolution Reaction. *ACS Materials Letters* **2019,** *1* (5), 526-533.

9. Fang, G.; Gao, J.; Lv, J.; Jia, H.; Li, H.; Liu, W.; Xie, G.; Chen, Z.; Huang, Y.; Yuan, Q.; Liu, X.; Lin, X.; Sun, S.; Qiu, H.-J., Multi-component nanoporous alloy/(oxy)hydroxide for bifunctional oxygen electrocatalysis and rechargeable Zn-air batteries. *Applied Catalysis B: Environmental* **2020,** *268*, 118431.

10. Liu, Q.; Duan, G.; Matsunaga, T.; Koshizuka, S.; Sun, Z., A Particle Shifting Model for Least Square Moving Particle Semi-Implicit Method with Numerical Integration on Wall Boundary Meshes. *The Proceedings of The Computational Mechanics Conference* **2021,** *2021.34* (0), 125.

11. Cui, X.; Zhang, B.; Zeng, C.; Guo, S., Electrocatalytic activity of high-entropy alloys toward oxygen evolution reaction. *MRS Communications* **2018,** *8* (3), 1230-1235.

12. Zhao, X.; Xue, Z.; Chen, W.; Bai, X.; Shi, R.; Mu, T., Ambient fast, large-scale synthesis of entropy-stabilized metal–organic framework nanosheets for electrocatalytic oxygen evolution. *Journal of Materials Chemistry A* **2019,** *7* (46), 26238-26242.

13. Sharma, L.; Katiyar, N. K.; Parui, A.; Das, R.; Kumar, R.; Tiwary, C. S.; Singh, A. K.; Halder, A.; Biswas, K., Low-cost high entropy alloy (HEA) for high-efficiency oxygen evolution reaction (OER). *Nano Research* **2021,** *15* (6), 4799-4806.

14. Asghari Alamdari, A.; Jahangiri, H.; Yagci, M. B.; Igarashi, K.; Matsumoto, H.; Motallebzadeh, A.; Unal, U., Exploring the Role of Mo and Mn in Improving the OER and HER Performance of CoCuFeNi-Based High-Entropy Alloys. *ACS Applied Energy Materials* **2024,** *7* (6), 2423-2435.

15. He, R.; Yang, L.; Zhang, Y.; Jiang, D.; Lee, S.; Horta, S.; Liang, Z.; Lu, X.; Ostovari Moghaddam, A.; Li, J.; Ibáñez, M.; Xu, Y.; Zhou, Y.; Cabot, A., A 3d‐4d‐5d High Entropy Alloy as a Bifunctional Oxygen Catalyst for Robust Aqueous Zinc–Air Batteries. *Advanced Materials* **2023,** *35* (46), 2303719.

16. He, R.; Yang, L.; Zhang, Y.; Wang, X.; Lee, S.; Zhang, T.; Li, L.; Liang, Z.; Chen, J.; Li, J.; Ostovari Moghaddam, A.; Llorca, J.; Ibáñez, M.; Arbiol, J.; Xu, Y.; Cabot, A., A CrMnFeCoNi high entropy alloy boosting oxygen evolution/reduction reactions and zinc-air battery performance. *Energy Storage Materials* **2023,** *58*, 287-298.

17. Bian, H.; Wang, C.; Zhao, S.; Han, G.; Xie, G.; Qi, P.; Liu, X.; Zeng, Y.; Zhang, D.; Wang, P., Preparation of highly efficient high-entropy alloy catalysts with electrodeposition and corrosion engineering for OER electrocatalysis. *International Journal of Hydrogen Energy* **2024,** *57*, 651-659.

18. Wang, Z.; Zhang, C.; Zhang, Y.; Hu, J., Ultrasound-Assisted Synthesis of High-Entropy Materials for Enhanced Oxygen Evolution Electrocatalysis. *Metals* **2024,** *14* (4), 384.

19. Bian, H.; Qi, P.; Xie, G.; Liu, X.; Zeng, Y.; Zhang, D.; Wang, P., HEA-NiFeCuCoCe/NF through ultra-fast electrochemical self-reconstruction with high catalytic activity and corrosion resistance for seawater electrolysis. *Chemical Engineering Journal* **2023,** *477*, 147286.

20. Yang, P.; Jiang, Z.; Shi, Y.; Zhang, W.; Ren, X.; Liang, L.; Wang, M.; Zhu, K., Construction of high-entropy oxy/hydroxides with dense nanosheet array structures on FeCoNiCrMo electrode for efficient oxygen evolution reaction. *Applied Physics Letters* **2023,** *123* (9), 091602.

21. Jin, Z.; Lyu, J.; Zhao, Y.-L.; Li, H.; Lin, X.; Xie, G.; Liu, X.; Kai, J.-J.; Qiu, H.-J., Rugged High-Entropy Alloy Nanowires with in Situ Formed Surface Spinel Oxide As Highly Stable Electrocatalyst in Zn–Air Batteries. *ACS Materials Letters* **2020,** *2* (12), 1698-1706.

22. Lu, Y.; Huang, K.; Cao, X.; Zhang, L.; Wang, T.; Peng, D.; Zhang, B.; Liu, Z.; Wu, J.; Zhang, Y.; Chen, C.; Huang, Y., Atomically Dispersed Intrinsic Hollow Sites of M‐M1‐M (M1 = Pt, Ir; M = Fe, Co, Ni, Cu, Pt, Ir) on FeCoNiCuPtIr Nanocrystals Enabling Rapid Water Redox. *Advanced Functional Materials* **2022,** *32* (19), 2110645.

23. Huang, K.; Xia, J.; Lu, Y.; Zhang, B.; Shi, W.; Cao, X.; Zhang, X.; Woods, L. M.; Han, C.; Chen, C.; Wang, T.; Wu, J.; Huang, Y., Self‐Reconstructed Spinel Surface Structure Enabling the Long‐Term Stable Hydrogen Evolution Reaction/Oxygen Evolution Reaction Efficiency of FeCoNiRu High‐Entropy Alloyed Electrocatalyst. *Advanced Science* **2023,** *10* (14), 2300094.

24. Chen, Z.; Wen, J.; Wang, C.; Kang, X., Convex Cube‐Shaped Pt_34_Fe_5_Ni_20_Cu_31_Mo_9_Ru High Entropy Alloy Catalysts toward High‐Performance Multifunctional Electrocatalysis. *Small* **2022,** *18* (45), 2204255.
